# Human EWS-FLI protein recapitulates in Drosophila the neomorphic functions that induce Ewing sarcoma tumorigenesis

**DOI:** 10.1093/pnasnexus/pgac222

**Published:** 2022-10-06

**Authors:** Cristina Molnar, Jose Reina, Anastasia Herrero, Jan Peter Heinen, Victoria Méndiz, Sophie Bonnal, Manuel Irimia, María Sánchez-Jiménez, Sara Sánchez-Molina, Jaume Mora, Cayetano Gonzalez

**Affiliations:** Institute for Research in Biomedicine (IRB Barcelona), The Barcelona Institute of Science and Technology, Carrer Baldiri Reixac 10, 08028 Barcelona, Spain; Institute for Research in Biomedicine (IRB Barcelona), The Barcelona Institute of Science and Technology, Carrer Baldiri Reixac 10, 08028 Barcelona, Spain; Institute for Research in Biomedicine (IRB Barcelona), The Barcelona Institute of Science and Technology, Carrer Baldiri Reixac 10, 08028 Barcelona, Spain; Developmental Tumor Biology Laboratory, Institut de Recerca Sant Joan de Déu, Esplugues de Llobregat 08950 Barcelona, Spain; Institute for Research in Biomedicine (IRB Barcelona), The Barcelona Institute of Science and Technology, Carrer Baldiri Reixac 10, 08028 Barcelona, Spain; Institute for Research in Biomedicine (IRB Barcelona), The Barcelona Institute of Science and Technology, Carrer Baldiri Reixac 10, 08028 Barcelona, Spain; Centre for Genomic Regulation (CRG), The Barcelona Institute of Science and Technology, 08003 Barcelona, Spain; Universitat Pompeu Fabra (UPF), 08002 Barcelona, Spain; Centre for Genomic Regulation (CRG), The Barcelona Institute of Science and Technology, 08003 Barcelona, Spain; Universitat Pompeu Fabra (UPF), 08002 Barcelona, Spain; Institucio Catalana de Recerca i Estudis Avançats (ICREA), Pg Lluis Companys 23, 08010 Barcelona, Spain; Developmental Tumor Biology Laboratory, Institut de Recerca Sant Joan de Déu, Esplugues de Llobregat 08950 Barcelona, Spain; Pediatric Cancer Center Barcelona (PCCB), Hospital Sant Joan de Déu, Esplugues de Llobregat, 08950 Barcelona, Spain; Developmental Tumor Biology Laboratory, Institut de Recerca Sant Joan de Déu, Esplugues de Llobregat 08950 Barcelona, Spain; Pediatric Cancer Center Barcelona (PCCB), Hospital Sant Joan de Déu, Esplugues de Llobregat, 08950 Barcelona, Spain; Developmental Tumor Biology Laboratory, Institut de Recerca Sant Joan de Déu, Esplugues de Llobregat 08950 Barcelona, Spain; Pediatric Cancer Center Barcelona (PCCB), Hospital Sant Joan de Déu, Esplugues de Llobregat, 08950 Barcelona, Spain; Institute for Research in Biomedicine (IRB Barcelona), The Barcelona Institute of Science and Technology, Carrer Baldiri Reixac 10, 08028 Barcelona, Spain; Institucio Catalana de Recerca i Estudis Avançats (ICREA), Pg Lluis Companys 23, 08010 Barcelona, Spain

## Abstract

Ewing sarcoma (EwS) is a human malignant tumor typically driven by the Ewing sarcoma-Friend leukemia integration (EWS-FLI) fusion protein. A paucity of genetically modified animal models, partially owed to the high toxicity of EWS-FLI, hinders research on EwS. Here, we report a spontaneous mutant variant, EWS-FLI_1FS_, that circumvents the toxicity issue in Drosophila. Through proteomic and genomic analyses, we show that human EWS-FLI_1FS_ interacts with the Drosophila homologues of EWS-FLI human protein partners, including core subunits of chromatin remodeling complexes, the transcription machinery, and the spliceosome; brings about a massive dysregulation of transcription that affects a significant fraction of known targets of EWS-FLI in human cells; and modulates splicing. We also show that EWS-FLI_1FS_ performs in Drosophila the two major neomorphic activities that it is known to have in human cells: activation of transcription from GGAA microsatellites and out competition of ETS transcription factors. We conclude that EWS-FLI_1FS_ reproduces in Drosophila the known oncogenic activities of EWS-FLI that drive EwS tumorigenesis in humans. These results open up an unprecedented opportunity to investigate EWS-FLI’s oncogenic pathways in vivo in a genetically tractable organism.

Significance Statement Modeling Ewing sarcoma is challenging, since overexpression of Ewing sarcoma-Friend leukemia integration (EWS-FLI) induces lethality or developmental defects. We have constructed Drosophila transgenic lines that circumvent the toxicity issue and in which the human EWS-FLI oncogene recapitulates some of its key oncogenic effects. Our work showing that specific neomorphic functions of EWS-FLI can be realistically reproduced in genetically engineered fly models opens up an unprecedented opportunity to investigate EWS-FLI’s oncogenic pathways in vivo in this genetically tractable organism.

## Introduction

Ewing sarcoma (EwS) is an exclusively human, aggressive tumor, typically arising from bone and soft tissues, that is reported to be the second most common bone malignancy in children, adolescents and young adults (1). Current management of EwS relies on a combination of cytotoxic drugs, surgery, and radiotherapy.

EwS is a paradigm for solid tumor development after a single genetic rearrangement: more than 80% of patients carry a fusion between the transactivation domain of the FET family member EWS RNA Binding Protein 1 (EWSR1) gene and the DNA binding domain of the E26 transformation specific (ETS) domain transcription factor Friend leukemia integration 1 (FLI1). Fusions of EWSR1 exon 7 to either FLI1 exon 6 (EWSR1-FLI1 type 1) or exon 5 (EWSR1-FLI1 type 2), account for 60% and 25% of Ewing sarcoma-Friend leukemia integration (EWS-FLI) fusions, respectively (2). In more than 25% of EwS patients, the EWSR1-FLI1 fusion is the only detectable genetic event at diagnosis (3).

EWS-FLI is a pleiotropic oncoprotein with diverse neomorphic functions that subvert cell physiology at different levels. EWS-FLI reprograms gene expression by direct interaction with two types of DNA sequences: microsatellites made of GGAA repeats (GGAAμSats) and enhancers containing ETS consensus sequences. Upon binding to GGAAμSats, EWS-FLI acts as a pioneer factor that recruits chromatin remodeling proteins and transforms these normally quiescent genomic regions into neo-enhancers that trigger transcription of hundreds of otherwise silent genes (4–7). Upon binding to ETS consensus sequences, EWS-FLI displaces wild-type ETS transcription factors, hence dysregulating the expression of their target genes (7, 8). In addition, through its interaction with core subunits of the spliceosome EWS-FLI changes the ratio of protein variants produced by alternative splicing (9). These neomorphic functions are at the core of EWS-FLI’s tumorigenic effect and are therefore well suited for targeted therapy.

There are many patient-derived EwS cell lines as well as human and mice cells engineered to express EWS-FLI, some of which have been used to develop xenograft and allograft models in mice and fish [reviewed in ref. (10)]. However, reproducing EwS in genetically modified transgenic animals has proven difficult. In mice, EWS-FLI expression driven by the Prx1 promoter results in abnormal muscle, cartilage, and bone development, but no tumors (11), while EWS-FLI expression driven from Osterix or Mx1 results in erythroid/myeloid leukemias, but not sarcomas (12, 13). In zebrafish, a first model based on transposon-mediated expression of EWS-FLI presented malignant peripheral nerve sheath tumors, and less frequently diffuse leukemia-like and small-round-blue-cell tumors (SRBCTs), but only in p53 deficient embryos (14). Recently, a new model based on Cre-inducible expression of ubi: GFP-2A-EWS-FLI in wild type zebrafish has been reported that causes rapid onset at high penetrance of SRBCTs that express canonical EWS-FLI target genes (15). Interestingly, there seems to be no spontaneous occurrence of EwS in mice or any other species, but humans (12, 16, 17).

Prompted by the need for genetically tractable experimental in-vivo models of the disease, we decided to investigate the effect of EWS-FLI in Drosophila. Anatomical and physiological differences between humans and Drosophila preclude the development of a realistic genetically modified model of EwS-like tumors in flies. However, given the remarkable conservation of molecular mechanisms and genes between Drosophila and humans (18–20), we hypothesized that the human EWS-FLI protein might recapitulate in flies at least some of the neomorphic functions that fuel EwS. If so, genetically modified Drosophila strains could provide a much needed genetically tractable experimental model to investigate EWS-FLI function.

## Results and discussion

### The human EWS-FLI fusion protein has a strong lethal effect in Drosophila

**Fig. 1.**
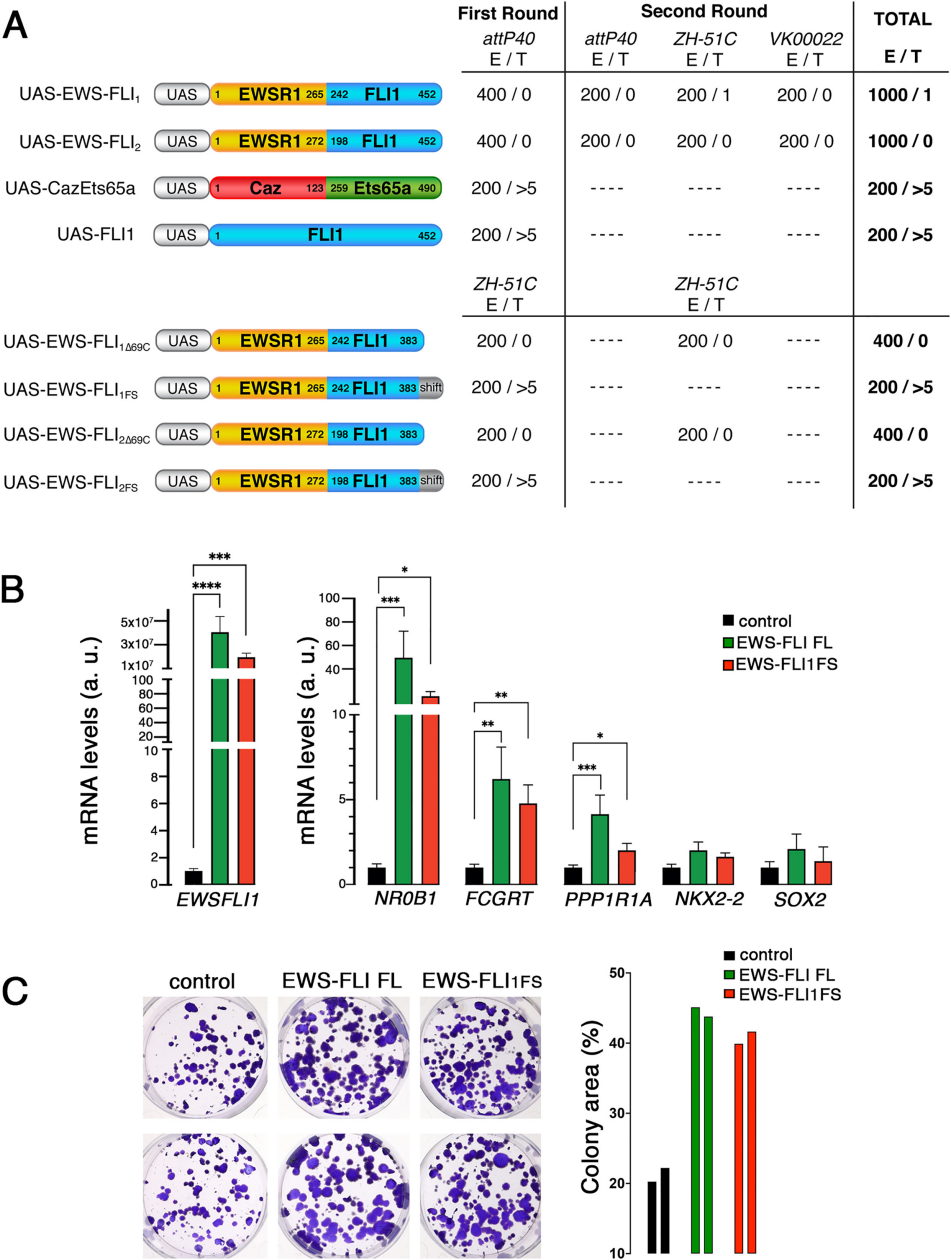
The EWS-FLI_1FS_ variant rescues the high toxicity of EWS-FLI in Drosophila. Number of injected embryos “E” and transgenic animals recovered “T” in two rounds of injection using attP40, ZH-51C, and VK00022 landing sites. (A) UAS transgenes carrying the human fusion proteins EWS-FLI Type 1 (UAS-EWS-FLI_1_), EWS-FLI Type 2 (UAS-EWS-FLI_2_), the homologue Drosophila fusion protein Caz-Ets65a (UAS-Caz-Ets65a), human FLI1 (UAS-FLI1), EWS-FLI Types 1 and 2 carrying a C-terminal 69 amino acid deletion (UAS-EWS-FLI_1∆69C_ and UAS-EWS-FLI_2∆69C_), or the C-terminal frame shift (UAS-EWS-FLI_1FS_ and UAS-EWS-FLI_2FS_). (B) Quantification by RT-qPCR of selected EWS-FLI target genes in HEK293 cells transfected with full-length EWS-FLI and EWS-FLI_1FS_. Error bars indicate SEM of data derived from three biological replicates (two technical duplicates each). a. u., arbitrary units. *P < 0.05; **P < 0.01; ***P < 0.001; ^****^P < 0.0001. (C) Clonogenic assay of control (empty vector), and stable full-length EWS-FLI and EWS-FLI_1FS_ expressing HEK293T cell lines, and quantitative analysis of colony area.

As a first step towards developing a Drosophila model capable of recapitulating the oncogenic functions of the human EWS-FLI fusion protein, we tried to generate transgenic fly strains carrying the coding sequences for EWS-FLI Type 1 and Type 2 (i.e. EWS-FLI_1_ and EWS-FLI_2_) under the control of the yeast Upstream Activating Sequence (UAS). We also generated strains carrying a similar fusion between Cabeza (Caz) and Ets65A, the Drosophila orthologs of EWSR1 and FLI, respectively (Fig. 1A).

Unlike other transgenic lines that were generated at the standard rate (i.e. > 5 per 200 injected embryos), no EWS-FLI_1_ nor EWS-FLI_2_ transgenic flies were recovered from 800 injected embryos (400 embryos each) (Fig. 1). These results strongly suggested that both Type 1 and Type 2 EWS-FLI fusion constructs must be toxic even at the exceedingly low rate of expression that UAS transgenes can be expected to leak in the absence of Gal4. Such toxicity levels have only been observed in UAS constructs encoding very potent cell lethal proteins like Ricin A or Diphtheria Toxin A [reviewed in ref. (21)].

To circumvent this problem, we carried out a second round of injection using different landing sites and at low temperature (18°C) to minimize leakiness. A total of 1200 embryos were injected. Once more, we failed to recover any UAS-EWS-FLI_2_ transgenic flies, but we did recover one fly from embryos injected with the UAS-EWS-FLI_1_ transgene (Fig. 1A). Notably, despite the difficulty of generating this fly, its offspring was fully viable and fertile and a stable line carrying the transgene was easily made.

DNA sequencing revealed that the transgene present in this line had a deletion of 17 nucleotides that causes the loss of six amino acids and a frameshift. The resulting protein is identical to EWS-FLI from amino acids 1 to 406 but lacks the 69 amino acid C-terminal tail of EWS-FLI that is replaced by a new 64 amino acid sequence. The entire EWSR1 portion as well as most of the FLI portion of EWS-FLI, including its entire DNA binding domain, remain, therefore, unaffected in this mutant that we have named EWS-FLI_1FS_ (Fig. 1A).

These results suggest that the strong toxicity of EWS-FLI transgenes in Drosophila maps to the 69 amino acid C-terminal tail that is lost in EWS-FLI_1FS_. To test this possibility, we first investigated the viability of full-length hFLI1 transgenes, which indeed carry the same C-terminal tail. We found that full-length hFLI1 transgenes can be recovered at the standard rate (>5 per 200 embryos) thus showing that the 69 amino acid C-terminal tail is not necessarily toxic in flies (Fig. 1). We then quantified the rate of recovery of transgenic lines carrying two different mutant versions of EWS-FLI1_1_ and EWS-FLI_2_. The first had the same genetic lesion found in EWS-FLI_1FS_. The second had a deletion of the 69 C-terminal amino acids. No transgenic flies carrying this deletion were recovered from a total of 800 injected embryos (Fig. 1, EWS-FLI_1Δ69C_ and EWS-FLI_2Δ69C_). However, both EWS-FLI_1FS_ and EWS-FLI_2FS_ transgenic lines carrying the frame shift version were recovered at the standard rate (Fig. 1A, EWS-FLI_1FS_ and EWS-FLI_2FS_). These results demonstrate that the lower toxicity of EWS-FLI_1FS_ is caused by replacing the 69 amino acid C-terminal peptide by the one that results from the EWS-FLI_1FS_ frameshift (Fig. 1A).

The same results were obtained regarding similar protein fusions that are less frequent but equally pathognomonic of EwS like EWS-ERG, EWS-FEV, and FUS-ERG in which the EWSR1 or FLI1 portions are substituted by the corresponding sequences of other members of the FET protein family like FUS RNA binding protein or other ETS transcription factors like ERG and FEV. The full-length and the corresponding C-terminal deletion versions of these fusion proteins appear to be as toxic as EWS-FLI, and only the frameshift versions can be recovered as transgenes (Fig. S1).

### The EWS-FLI_1FS_ variant retains neomorphic functions of full-length EWS-FLI in human cells

Work on NIH3T3 mouse fibroblasts suggested a role for the C-terminal region of FLI in  mediating transcriptional down-regulation by EWS-FLI (22). However, later functional studies on the effect of an 81 C-terminal EWS-FLI deletion ruled out any significant role for this region in oncogenic transformation in an EwS cellular context (23). These results strongly suggest that EWS-FLI_1FS_, which is only lacking the 69 C-terminal amino acids might also retain the neomorphic functions of full-length EWS-FLI. To test this hypothesis, we tested EWS-FLI_1FS_’ effect on both gene expression and clonogenicity in human HEK293 cells. To determine the effect on gene expression, we selected a set of validated EWS-FLI target genes: *FCGRT, NR0B1, NKX2-2, PPP1R1A*, and *SOX2* (4, 24–26). We found that like full-length EWS-FLI, EWS-FLI_1FS_ significantly upregulates *NR0B1, PPP1R1A*, and *FCGRT* (Fig. 1B). Neither EWS-FLI_1FS_ nor full-length EWS-FLI had a significant effect on the expression of *NKX2-2* and *SOX2*. We do not know why wild-type EWS-FLI fails to upregulate *NKX2-2* and *SOX2* in HEK293, but it may reflect the fact that out of the five selected genes these are the two whose transcription start sites are furthest away for the GGAA repeats (63 Kb for *NKX2-2* and 470 Kb for *SOX2*). It is thus possible that transcriptional rewiring of these genes from such distant enhancers may take longer than 72 h post transfection with EWS-FLI in HEK293 cells.

These results show that EWS-FLI_1FS_ retains full-length EWS-FLI’s neomorphic capability to upregulate three Ewing sarcoma signature genes in HEK293 cells. We also found EWS-FLI_1FS_ and full-length EWS-FLI to be equally competent at significantly increasing clonogenicity in HEK293T cells (Fig. 1C). Altogether, these results are consistent with the hypothesis that EWS-FLI_1FS_ retains the neomorphic functions of full-length EWS-FLI.

### EWS-FLI_1FS_ brings about a massive rewiring of the transcriptome in Drosophila

EWS-FLI brings about a major dysregulation of transcription in human cells. To determine if EWS-FLI_1FS_ may have similar effects in Drosophila, we undertook a genome-wide transcriptome analysis using Affymetrix. To identify a suitable cell type, we expressed EWS-FLI_1FS_ from a variety of Gal4 drivers. We found that EWS-FLI_1FS_ expression from most of these Gal4 drivers had strong deleterious effects and caused embryonic lethality (Table S1), thus showing that the EWS-FLI_1FS_ variant remains highly toxic. Indeed, leaky expression (in the absence of any Gal4 driver) of two copies of the UAS-EWS-FLI_1FS_ transgene is also lethal. These results are consistent with previous reports showing that EWS-FLI is not permissive in most cell types in human, mice, and fish (27, 28).

One of the drivers that allowed for larval development was *P{GawB}nubbin-AC-62* (henceforth referred to as *nub-Gal4*). Although generally used for its prominent expression in the wing pouch of the wing imaginal disc (29), *nub-Gal4* also drives Gal4 expression in the salivary glands.

Remarkably, expression of EWS-FLI_1FS_ from the *nub-Gal4* driver in the wing disc epithelia results in tumors that present immortal malignant neoplastic growth in allograft assays (Fig. S2). These data strongly substantiate the tumorigenic potential of the EWS-FLI fusion by itself, without the concourse of other genetic lesions. Importantly, however, these epithelia-derived tumors bear little resemblance to EwS. We, therefore, worried that tumor type-specific changes in their transcriptome could overshadow those specifically induced by EWS-FLI, which are the ones we wished to identify. To circumvent this problem, we focused our attention on EWS-FLI expressing salivary glands that appear to develop fairly normally.

**Fig. 2.**
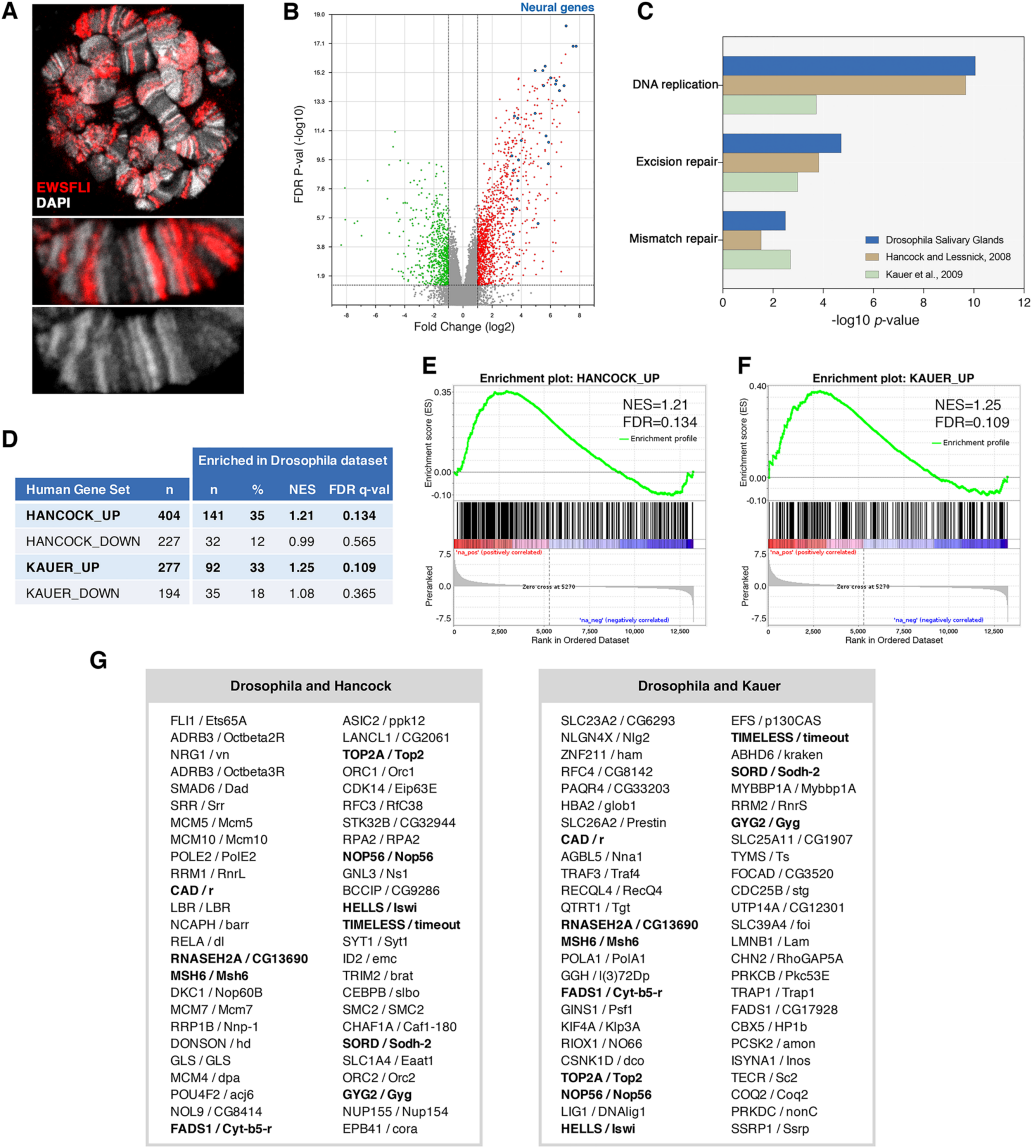
EWS-FLI_1FS_ brings about a massive rewiring of the transcriptome in Drosophila salivary glands. (A) Polytene chromosomes of *nub-Gal4/UAS-EWS-FLI_1FS_* salivary glands, stained with anti-EWS antibody (red) and DAPI (grey). The lower panels show higher magnification views. (B) Volcano plot showing changes in gene expression levels in EWS-FLI_1FS_ (*nub-Gal4/UAS-EWS-FLI_1FS_*) compared to control (*nub-Gal4/+*) samples. Red and green dots represent genes that are significantly (FDR = 0.05) up (FC > 2) and downregulated (FC< -2) genes, respectively; blue dots represent strongly upregulated (FC > 10) neural genes. (C) Bar graph showing significantly enriched KEGG pathways of upregulated genes in EWS-FLI Drosophila salivary glands (blue bars), in Hancock and Lessnick, 2008 (brown bars), and in Kauer et al., 2009 (green bars). (D) Gene Set Enrichment Analysis (GSEA) of EWS-FLI_1FS_ salivary glands ranked dataset compared with the Hancock and Lessnick, 2008 and the Kauer et al., 2009 signatures. Significantly enriched gene sets are shown in bold (FDR < 0.25). (E and F) GSEA plots of upregulated genes in Hancock and Lessnick, 2008 (E) and in Kauer et al., 2009 (F). (G) Lists of the first 50 genes in the ranked list that significantly contribute to the enrichment of each gene set. Common genes are shown in bold.

During larval development, Drosophila salivary glands’ secretory cells undergo multiple rounds of DNA synthesis without mitosis (i.e. endoreduplication). The resulting so called polytene chromosomes are made of hundreds of chromatids aligned to one another along their entire length and display a reproducible pattern of bands and interbands that correlate tightly with chromosome structure and transcriptional activity [reviewed in ref. (30)]. Immunofluorescence with an anti-EWSR1 antibody on nub >EWS-FLI_1FS_ salivary glands showed that EWS-FLI_1FS_ binds to polytene chromosomes in a banding pattern that largely overlaps DAPI interbands and loosely condensed bands (Fig. 2A), which correspond to transcriptionally active genes (30).

Affymetrix profiling showed a massive change of the salivary glands’ transcriptome upon *nub-Gal4*-driven expression of EWS-FLI_1FS_ with a total of 1021 and 488 genes significantly up (FC > 2) and downregulated (FC< -2), respectively (Fig. 2B, red and green dots; Table S2). In some cases, the extent of dysregulation is extreme like the 86 genes that have little or no expression in control salivary glands and are upregulated between 10 and 300-fold upon EWS-FLI_1FS_ expression. Notably, a third (*n* = 32) of these genes are associated with neural development (Fig. 2B, blue dots; Fig. S3). Upregulation of neural genes is a common trait of EwS cells and EWS-FLI has been shown to promote an Ewing-specific neuroectodermal gene expression program in a wide variety of cell types (31–34). To validate the Affymetrix data, the expression levels of nine up and eight downregulated genes in EWS-FLI_1FS_ and control salivary glands were confirmed by RT-qPCR (Fig. S4 and Table S3).

We then compared our results with published transcriptomic signatures derived from two meta-analyses of dozens of EwS’s cellular models and tumor samples (35, 36). To this end, we identified enriched KEGG pathways and carried out gene set enrichment analysis (GSEA) (37, 38). We found eight KEGG pathways that are significantly enriched (*P* < 0.05) in our EWS-FLI salivary gland signature, 18 in Hancock and Lessnick, 2008 and 11 in Kauer et al., 2009. Notably, DNA replication, excision repair, and mismatch repair, which are the most significantly enriched KEGG pathways in our EWS-FLI_1FS_ salivary gland signature, are also among the most significantly enriched KEGG pathways in the published human signatures (Fig. 2C).

GSEA revealed that as far as upregulated genes are concerned, a third of both the Hancock and Lessnick, 2008, (141/404) and the Kauer et al., 2009 (92/277) signatures are significantly enriched in the top of our GSEA-ranked Drosophila dataset with FDR *q*-values of 0.134 and 0.109, respectively (Fig. 2D to F). The 50 genes with the highest fold change in the Drosophila dataset that are also present in both human gene sets are shown in Fig. 2G. Included among these are some well-known targets of EWS-FLI in EwS cells like *ID2* (*emc*), *FLI* (*Ets65A* and *Ets21C*), *TOP2A* (*Top2*), and *TIMELESS* (*Timeout*).

Other orthologs of known targets of EWS-FLI in EwS cells like *LOX, FOXO1, GLI1*, or *NKX2.2* were not dysregulated in our samples. However, differences in EWS-FLI-driven gene expression dysregulation are notorious even among datasets derived from human cells as substantiated by the partial overlap between different transcriptomics studies (35, 36). Interestingly, some validated targets of EWS-FLI in EwS like *IGF1* (39) which are not included in the Hancock and Lessnick, 2008 and Kauer et al., 2009 signatures are top-ranking in the Drosophila signature.

Downregulated gene signatures from the Hancock and Lessnick, 2008, and Kauer et al., 2009, were not significantly enriched in the EWS-FLI_1FS_ Drosophila transcriptome. A poorer extent of overlap in downregulated versus upregulated genes among datasets from different human and murine EwS model systems has been reported before (35, 36). This is likely to result from the fact that upregulation can affect any gene regardless of the cell type in which it is normally expressed, while only genes that are expressed in the cell type under study can be downregulated (35). Downregulation of the existing cell differentiation program is a common trait of cellular EwS models (31, 32). Consistently, a fifth (21/101) of the most downregulated genes (FC< -5) in our Drosophila dataset are salivary gland-specific genes.

These results reveal that expression of EWS-FLI_1FS_ in Drosophila brings about a major dysregulation of gene transcription that affects hundreds of genes, including a significant fraction of known targets of EWS-FLI in EwS.

### The EWS-FLI_1FS_ fusion interacts with Drosophila subunits of RNApol II, chromatin remodeling complexes and the spliceosome

**Fig. 3.**
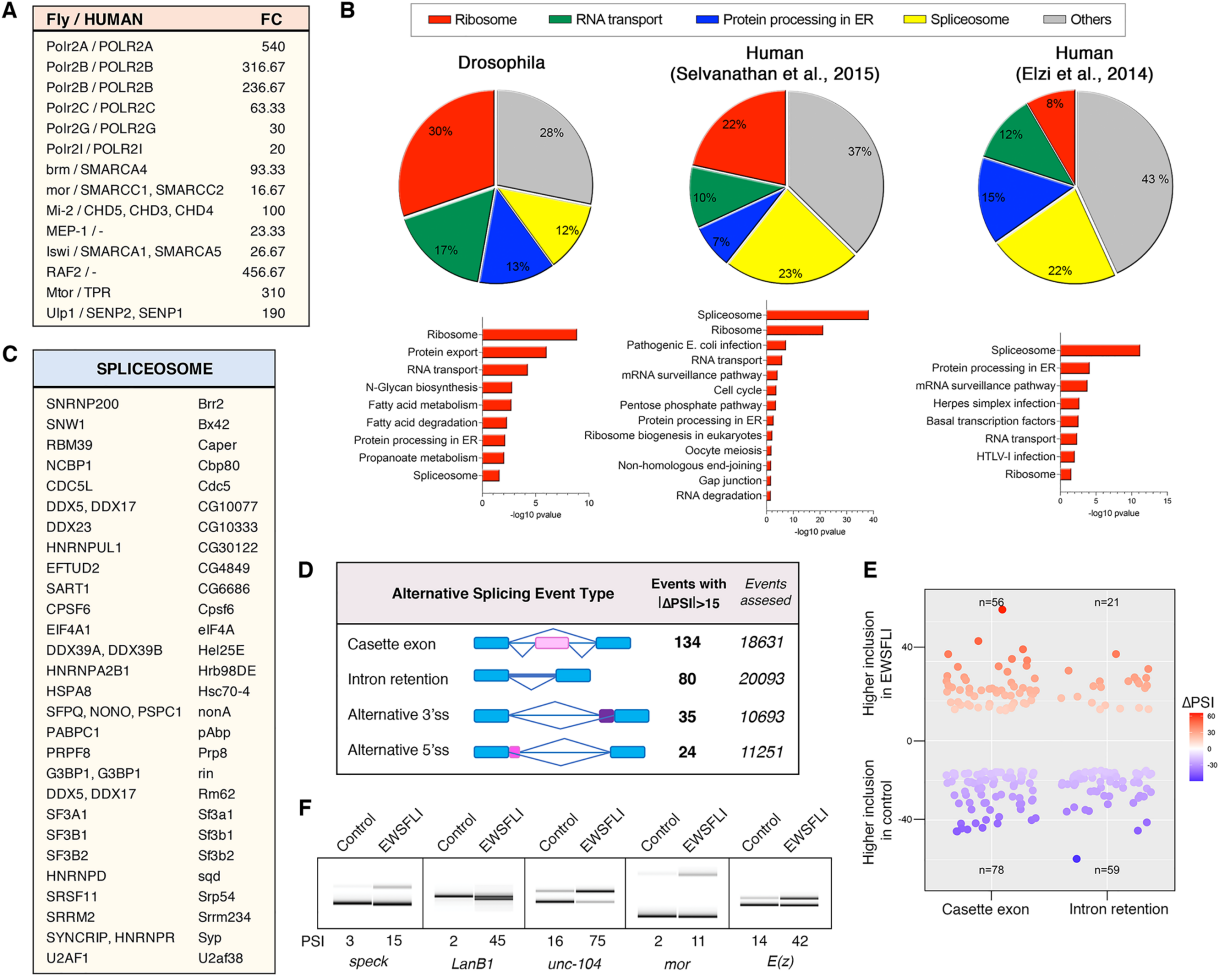
EWS-FLI_1FS_ interacts with subunits of RNApol II, chromatin remodeling complexes and the spliceosome and modulates splicing in Drosophila. (A) Selected, top ranking interactors of Drosophila EWS-FLI_1FS_ with their corresponding human orthologs and Fold Change (FC) between EWS-FLI_1FS_ (*nub-Gal4/UAS-EWS-FLI_1FS_*) and control (*nub-Gal4/+*) samples. (B) Pie graphs showing the most enriched KEGG pathways identified in EWS-FLI_1FS_ Drosophila interactome and in two published human interactomes (9, 50). Bar graphs show the corresponding *P*-values (-log10). (C) Spliceosome proteins identified as EWS-FLI interactors in Drosophila salivary glands and in the Selvanathan et al. and Elzi et al. studies. (D) Alternative splicing (AS) events driven by EWS-FLI_1FS_ in Drosophila salivary glands as inferred from RNA-seq analyses showing the type and number of events with absolute ∆PSI > 15. (E) Plot representing ∆PSI values (*y*-axis) of cassette exon and intron retention events (*x*-axis) differentially spliced between EWS-FLI_1FS_ and control samples. The upper part corresponds to events with higher inclusion in EWS-FLI_1FS_ salivary glands, and the lower part to events with higher inclusion in control samples. (F) Validation by RT-PCR of selected AS changes.

EWS-FLI’s oncogenic functions depend upon its direct interaction with specific proteins. To identify the interactome of EWS-FLI_1FS_ in Drosophila, we carried out co-immunoprecipitation (co-IP) with an anti-EWSR1 antibody followed by mass spectrometry (MS). We identified 631 proteins significantly enriched (FC > 3 and BFDR < 0.02) in nub > EWS-FLI_1FS_ salivary gland cells compared to control samples. A total of 594 of these proteins map to human orthologs. Top ranking in terms enrichment are some of the core components of transcription and ATP-dependent chromatin remodeling complexes that are known to interact with, and be required for, the oncogenic function of EWS-FLI in human cells (Fig. 3A).

Polr2A, Polr2B, Polr2C, Pol2rG, and Polr2I are subunits of the RNA polymerase holoenzyme II (RNA pol II), the synthetic apparatus of mRNA. The human orthologue of dPol2rG, RBP7, is one of the first and best characterized EWS-FLI interacting proteins in EwS cells. Pol II binding to EWS-FLI is a key step in the transcriptional upregulation of EwS signature genes (40–42). Brahma (Brm) and Moira (Mor) are core components of the Drosophila Brahma associated proteins (BAP) complex and orthologs of the equivalent human BRG1/BRM–associated factor (BAF) complex components SMARCA4 (a.k.a BRG1) and SMARCC1/SMARCC2. These, and other BAF complex proteins, have been shown to co-purify with EWS-FLI in human cells (9, 43) and their recruitment to tumor-specific enhancers is a key triggering event in EwS oncogenesis (44). Mi-2 and MEP-1 are components of the Drosophila nucleosome remodeling and deacetylase (NurD) complex. Mi-2 is the ortholog of human CHD3, CHD4, and CHD5 that are structural components of the human NurD complex, which has been shown to mediate transcriptional repression in EwS (45). Human NurD complex proteins CHD4, HDAC2, and RBBP7 co-purify with EWS-FLI from EwS cell extracts (9). Imitation SWI (Iswi), the orthologue of human SMARCA1 and SMARCA5, is the core component of the Drosophila ISWI family complexes (46, 47). There are no published data linking ISWI with EwS development, but SMARCA5, and four other proteins of human ISWI have been shown to co-purify with EWS-FLI from human cells extracts (9).

Other highly enriched proteins in our EWS-FLI_1FS_ co-IP Drosophila dataset are RING-associated factor 2 (RAF2), Megator (Mtor/TPR), and Ulp1(SENP2). RAF2, MTOR, and ULP1 co-elute as a large multiprotein assemblage with the core Drosophila Polycomb group (PcG) complex dRING-associated factors (dRAF), which includes Sce/hRING1B, PSC/hBMI1, and dKDM2/hKMD2 (48). Raf2 is a direct interactor of Sce/hRINGB in Drosophila (48) and its human orthologue RING1B is a critical modulator of EWSR1-FLI1–induced chromatin remodeling in EwS (49). TPR, the human homologue of Drosophila Mtor was also found to co-purify with EWS-FLI in human cells (9).

To further investigate the resemblance between the EWS-FLI_1FS_ Drosophila interactome with that of EWS-FLI in human cells, we identified enriched KEGG pathways and gene ontologies in our dataset and in two published studies of the human EWS-FLI interactome (9, 50). Remarkably, in all three datasets more than half of the proteins assigned to KEGG pathways belong to one of only four KEGG categories: “ribosome,” “spliceosome,” “RNA transport,” and “protein processing in ER” (Fig. 3B). The Selvanathan et al., 2015 study identified 14 proteins belonging to the “protein processing in ER,” five of which are orthologs of proteins enriched in our Drosophila samples. KEGG categories “ribosome” and “RNA transport” include 67 ribosomal subunits and 11 eukaryotic initiation factors, many of which are orthologs of human genes identified in the Selvanathan et al., 2015 and Elzi et al., 2015 studies. The Drosophila “spliceosome” category includes 41 proteins of which 28 are orthologs of human proteins identified as EWS-FLI interactors in the Selvanathan et al., study (Fig. 3C). Five of these were also found in the Elzi et al., study. These results demonstrate that EWS-FLI_1FS_ is capable of interacting with the Drosophila orthologues of proteins with which EWS-FLI interacts in EwS cells, including subunits of RNApol II, chromatin remodeling complexes and the spliceosome that are essential for EWS-FLI tumorigenic effect (44, 45).

### EWS-FLI_1FS_ modulates splicing in Drosophila

Alternative splicing (AS) increases the functional diversity of proteins and noncoding RNAs and is key in the regulation of many cellular processes (51). In human cells, EWS-FLI modulates AS through its interaction with essential splicing factors like PRPF6, PRPF8, PRPF9, DDX5, SF1, and U1C (9, 52). Conversely, some splicing factors have been shown to modulate the transcriptional activity of EWS-FLI (53). Prompted by the abundance of spliceosome subunits in the EWS-FLI_1FS_ interactome, including core subunits of the pre-mRNA splicing complex like Prp8 and Prp19 (54, 55), we decided to investigate if EWS-FLI_1FS_ might also modulate splicing in Drosophila. To this end, we generated RNA-seq data and quantified differences in AS profiles between EWS-FLI_1FS_-expressing and control salivary glands using *vast-tools* (56, 57).

We found 273 alternative events with a ∆PSI (delta Percent Spliced In) ≥ 15 between EWS-FLI_1FS_ expressing and control salivary glands. A study in EwS cell line TC32 identified 386 AS events dependent upon EWS-FLI. Taking into account that there are on average 4 introns per gene in Drosophila (Flybase, FB2021_04 Release Notes) and 8 to 9 in human (58), the number of AS events caused by EWS-FLI_1FS_ that we have found in Drosophila is in relative terms similar to that reported in the TC32 cell line (9).

EWS-FLI_1FS_-driven AS events in Drosophila salivary glands included cassette exons, intron retention, and alternative 3’ and 5’ splice sites (Fig. 3D). Interestingly, among regulated introns, EWS-FLI_1FS_-driven enhanced removal (*n* = 59) outnumbered enhanced retention (*n* = 21), hence suggesting that EWS-FLI_1FS_ activity can be rate limiting for removal of certain introns (Fig. 3D and E). On the other hand, EWS-FLI_1FS_ induced skipping of 78 cassette exons, while promoted inclusion of 56, indicating that EWS-FLI_1FS_ can act either as an activator or as a repressor of alternatively spliced exons (Fig. 3D and E). Similar effects were observed in EwS TC32 cells in which retained introns almost doubled and skipped exons dropped upon EWS-FLI depletion (9). The results derived from the bioinformatics analysis using *vast-tools* were validated by RT-PCR for all five genes chosen for this purpose (Fig. 3F and S5). Sashimi plots of these splicing events are shown in Fig. S6. Included among them are *mor* and *E(z)* whose orthologs are also alternatively spliced in human EwS TC32 cells (9).

These results show that, as in EwS cells, EWS-FLI_1FS_ interaction with the spliceosome modulates splicing in Drosophila.

### EWS-FLI_1FS_ dysregulates ETS domain transcription factors’ targets

**Fig. 4.**
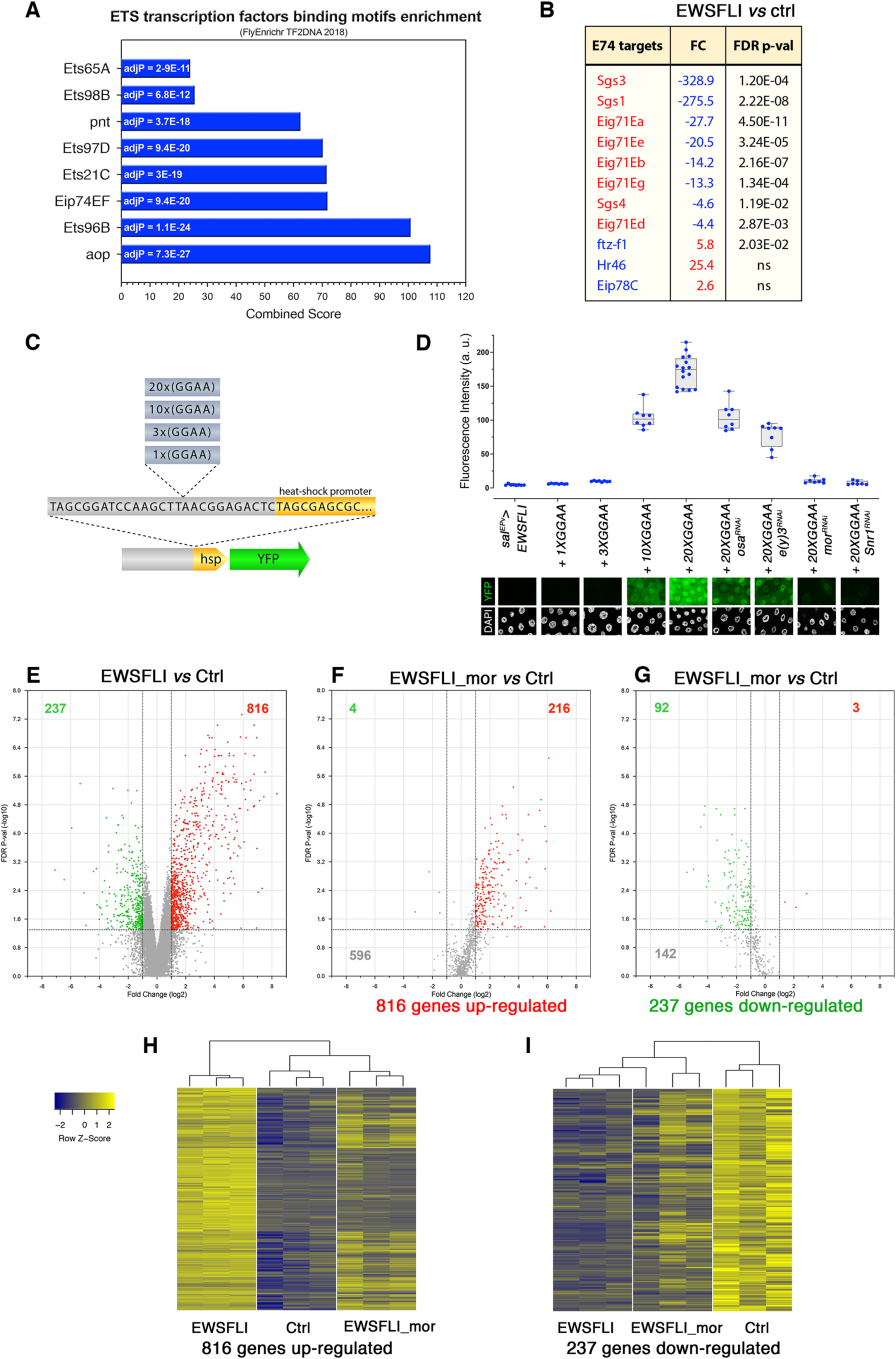
EWS-FLI_1FS_ outcompetes ETS transcription factors and drives BAP complex-dependent transcription from GGAAμSat sequences. Bar graph representing the enrichment (adjusted *P*-value and combined score) of ETS transcription factors’ regulated genes in the EWS-FLI_1FS_ Drosophila signature. (B) EWS-FLI_1FS_-induced dysregulation of genes that are positively (red) and negatively (blue) regulated by E74. The corresponding FC values are in blue for genes that are downregulated by EWS-FLI_1FS_, and in red for genes that are upregulated by EWS-FLI_1FS_. (C) Cartoon representing the GGAAµSat constructs engineered to generate fly transgenic strains carrying 1, 3, 10, and 20 GGAA repeats upstream a minimal *hsp70* promoter and followed by the YFP coding sequence. (D) Quantification of YFP fluorescence in salivary glands carrying GGAAµSat > YFP constructs and expressing EWS-FLI_1FS_. YFP signal is not detected in salivary glands carrying *1X(GGAA)µSat-YFP* and *3X(GGAA)µSat-YFP*, but is strong in salivary glands carrying 10x(GGAA)μSat > YFP and 20x(GGAA)μSat > YFP. YFP fluorescence from the 20x(GGAA)μSat > YFP transgene is diminished upon RNAi depletion of the BAP chromatin complex components *osa, e(y)3, mor* and *Snr1*. Representative examples of salivary glands micrographs are shown below. a.u., arbitrary units. (E to G) Volcano plots showing changes in gene expression levels between EWS-FLI_1FS_ (*sal^EPv^-Gal4/UAS-EWS-FLI_1FS_*) and control (Ctrl; *sal^EPv^-Gal4/+*) samples (E) and the effect of loss of *mor* on EWS-FLI_1FS_-dependent upregulated (F) and downregulated (G) genes. Red and green dots represent up (FC > 2) and downregulated (FC< -2) genes, respectively. (H and I) Heatmaps of gene expression profiles of control (Ctrl; *sal^EPv^-Gal4/+*), EWS-FLI (*sal^EPv^-Gal4/UAS-EWS-FLI_1FS_*), and EWS-FLI_mor (*sal^EPv^-Gal4 UAS-EWS-FLI_1FS_/mor^RNAi^*) salivary glands. Probesets correspond to genes significantly up (H) or downregulated (I) in EWS-FLI_1FS_ compared to Ctrl. Expression levels are reported as Row Z-score; blue and yellow indicate low and high expression, respectively. Dendrograms on the top of the heatmaps show hierarchical clustering between samples.

EWS-FLI interferes with enhancers containing canonical ETS motifs by displacing wild-type ETS transcription factors, hence disrupting the expression of ETS target genes (7). To assess the effect of EWS-FLI_1FS_ expression on ETS target genes in Drosophila salivary glands, we ran the EWS-FLI_1FS_ transcriptomic signature (Table S2) against the TF2DNA 2018 database of transcription factor binding motifs and regulated genes using FlyEnrichr (59, 60). We found that all eight ETS domain transcription factors of Drosophila score very high, with adjusted *P*-values < 1.0E−10 and combined scores between 20 and 110 (Fig. 4A).

One of the ETS domain transcription factors that is most significantly affected is E74, which is encoded by the Eip74EF gene (adj-*P* value = 9.4E−20; combined score = 71). E74 is essential for the regulation of many ecdysone secondary-response genes. In salivary glands, there are eight and three known targets genes for which E74 is a positive and negative regulator, respectively (61, 62). Remarkably, most of these genes are strongly dysregulated upon EWS-FLI_1FS_ expression (Fig. 4B). These results underpin the complexity of the mechanisms through which EWS-FLI rewires the transcriptome and suggest that, as in human cells, EWS-FLI is able to outcompete natural ETS transcription factors in Drosophila (7).

### EWS-FLI_1FS_ drives BAP-dependent transcription from GGAAμSat sequences

BAP complex-dependent conversion of silent GGAAμSats into upstream activating sequences or neo-enhancers accounts for a significant fraction of the genes that are upregulated by EWS-FLI in EwS. GGAAμSats of around 20 GGAA repeats are best suited as EWS-FLI responsive elements (4, 5, 7, 44, 63).

Our results showing that EWS-FLI_1FS_ co-immunoprecipitates with subunits of RNApol Il and chromatin remodeling complexes, including BAP/BAF itself, suggest that this fusion protein may also be capable of driving transcription from GGAAμSats in Drosophila. However, in stark contrast with the human genome that contains thousands of GGAAμSats in the range between 18 and 22 GGAA repeats in length (64), GGAAμSats are extremely rare and much shorter in Drosophila: the r6.40 reference genome contains only 83 GGAAμSats longer than 2 GGAA consecutive repeats and the longest one in the entire genome has only 9 GGAA repeats.

To make it possible, to test if the EWS-FLI_1FS_ fusion can trigger the chain of events that activate transcription from GGAAμSats in Drosophila, we generated a new set of genetically engineered fly models carrying GGAAμSats that had 1, 3, 10, and 20 GGAA repeats upstream the heat shock minimal promoter followed by the YFP coding sequence (Fig. 4C). For technical reasons, these experiments were carried out driving EWS-FLI_1FS_ transcription from *sal^EPv^-Gal4*, that like *nub-Gal4* is also expressed in the salivary glands. We observed no YFP signal upon EWS-FLI_1FS_ expression in salivary glands carrying 1x(GGAA)μSat > YFP or 3x(GGAA)μSat > YFP transgenes. However, YFP fluorescence levels were high, around 100 and 150-fold over background level, in salivary glands expressing EWS-FLI_1FS_ and carrying 10x(GGAA)μSat > YFP and 20x(GGAA)μSat > YFP transgenes, respectively (Fig. 4D). EWS-FLI_1FS_ can also upregulate YFP expression from the 20x(GGAA)μSat > YFP transgene in other tissues like wing discs (*sal^EPv^-Gal4*), eye discs (*GMR-Gal4)*, and the neuroepithelium (*ogre-Gal4*) (Fig. S7). In all these cases, development of the EWS-FLI_1FS_-expressing tissue is severely compromised.

Notably, the type 2 fusion EWS-FLI_2FS_ is equally capable of activating YFP transcription from the 20x(GGAA)μSat, and notably, so is the Caz-Ets65A fusion made between the Drosophila homologues of human EWSR1 (Caz) and FLI1 (Ets65A) although with a lower productivity than its human counterpart (Fig. S8).

To determine if as in EwS cells, EWS-FLI_1FS_-driven transcription from GGAAμSats requires the BAF chromatin remodeling complex in Drosophila, we quantified YFP fluorescence in salivary glands that in addition to carrying the 20x(GGAA)μSat > YFP transgene and expressing EWS-FLI_1FS_ were depleted for each of ten members of the BAP/BAF complex by RNAi. We found that depletion of either *mor* (Hs SMARCC1 and SMARCC2) or *Snr1* (Hs SMARCB1) essentially eliminates EWS-FLI-dependent YFP expression from the 20x(GGAA)μSat, which is also reduced to a greater or lesser extent by depletion of any of the ten components of the complex that we tested (Fig. 4D and S9).

These results demonstrate that EWS-FLI_1FS_ can productively interact with and convert transcriptionally silent GGAAμSats into neo-enhancers to bring about BAF/BAP-dependent unscheduled transcription in Drosophila cells. In addition, these results further substantiate the conclusion that the C-terminal region affected in EWS-FLI_1FS_ is not essential for driving transcription from GGAAμSats.

### The BAP/BAF complex is required for GGAAμSat-independent EWS-FLI_1FS_–driven dysregulation of the Drosophila transcriptome

Having found that BAF/BAP is essential for EWS-FLI-driven transcription from GGAAμSats we asked whether this chromatin remodeling complex might also be required for the massive rewiring of the transcriptome generated by EWS-FLI_1FS_ in salivary glands, which indeed cannot be accounted for by the few and short GGAAμSats that are present in the Drosophila genome.

To answer this question, we investigated the effect of loss of *mor* on the EWS-FLI_1FS_–dependent transcriptome. We found that nearly three quarters (73%, *n* = 596) of 816 genes that are upregulated in salivary glands expressing EWS-FLI_1FS_ from the *sal^EPv^-Gal4* driver are not significantly upregulated in salivary glands that in addition to EWS-FLI_1FS_ express *mor-RNAi* (Fig. 4E and F). Likewise, over half (60%, *n* = 142) of 237 genes that are downregulated by EWS-FLI_1FS_ are not in EWS-FLI_1FS_ expressing salivary glands that are depleted of *mor* (Fig. 4E and G). These results show that a large fraction of the EWS-FLI_1FS_ signature is dependent upon the Drosophila orthologue of human BAF HsSMARCC1 and SMARCC2 subunits. Consistent with these results, heat maps show EWS-FLI_1FS_ expressing samples to be clearly distinct from *mor*-depleted, EWS-FLI_1FS_ expressing samples both for genes upregulated and downregulated by EWS-FLI_1FS_ (Fig. 4H and I). However, hierarchical clustering shows *mor*-depleted EWS-FLI_1FS_ expressing samples to be closer to control for genes upregulated by EWS-FLI_1FS_, but closer to EWS-FLI_1FS_ expressing samples for downregulated genes (Fig. 4H and I, and Fig. S9B and C). These results strongly suggest that the BAP/BAF chromatin remodeling complex is necessary for the dysregulation of more than half of the EWS-FLI_1FS_ Drosophila signature, which, indeed, is GGAAμSat independent.

Altogether, we have found that the human EWS-FLI, EWS-ERG, EWS-FEV, and FUS-ERG fusion proteins are so strongly toxic to Drosophila that we could no generate lines carrying UAS transgenes encoding any of these oncogenes. However, we have identified a spontaneous mutant frameshift variant that circumvents this limitation thanks to the new C-terminal 64 amino acid tail.

Remarkably, human EWS-FLI_1FS_ can interact with the fly homologues of many of the EWS-FLI human protein partners that are core subunits of chromatin remodeling complexes, transcription machinery, and spliceosome. Through these interactions, EWS-FLI_1FS_ is able to recreate in flies the neomorphic functions that account for the EWS-FLI oncogenic effect in humans: a massive reshaping of the transcriptome affecting, among others, targets of all eight of the fly’s ETS transcription factors; de novo creation of enhancers at GGAAμSats; and changes of the ratio of protein variants produced by alternative splicing. In addition, EWS-FLI_1FS_ induces malignant tumors in the wing disc that, although epithelia-derived and different to EwS, are remarkable because they strongly substantiate the conclusion that EWS-FLI alone can trigger malignant growth in a living animal.

Anatomical and physiological differences preclude the generation in flies of tumors that reproduce the complexity of traits that define specific human cancer types. This is even more so in the case of EwS that stands out as the paradigm of cancer type that develops only in humans and for which even rodents have failed to provide realistic models. However, our work showing that specific neomorphic functions of EWS-FLI can be realistically reproduced in genetically engineered fly models opens up an unprecedented opportunity to investigate EWS-FLI function in vivo in this genetically tractable organism. Indeed, Drosophila has a solid track record as a model system and has made seminal contributions to our understanding of fundamental biology processes, including splicing, transcription, and chromatin remodeling. The high-content sophisticated functional assays that are standard in Drosophila research may help to unveil new paths of intervention to fight the deadly human disease.

## Materials and methods

Detailed materials and methods, including fly stocks, genotypes, crossing schemes, allograft assays, immunohistochemistry, cloning and generation of transgenic lines are provided in the Supplementary Material Appendix. Microarray processing was performed using the Drosophila Genome 2.0 protocol (Affymetrix/ThermoFisher Scientific), and scanned with a GeneChip Scanner GCS3000 (Affymetrix/ThermoFisher Scientific). Alternative splicing was quantified from RNA sequencing data obtained from salivary glands using the toolset vast-tools v2.5.1 (56) [dm6, VASTDB library: vastdb.dme.23.06.20.tar.gz (57)]. Samples for nano LC-MS/MS mass spectrometry were processed in an Orbitrap Fusion Lumos™ Tribrid. Detailed and fully referenced information on all these methods can be found in the Supplementary Material Appendix.

## Supplementary Material

pgac222_Supplemental_Files

## Data Availability

All data needed to evaluate the conclusion in the paper are present in the manuscript and/or the Supplementary Materials.

## References

[1] Riggi N , Suvà ML, Stamenkovic I. 2021. Ewing’s Sarcoma. N Engl J Med. 384:154–164. 33497548 10.1056/NEJMra2028910

[2] Zucman J et al. 1993. Combinatorial generation of variable fusion proteins in the Ewing family of tumours. EMBO J. 12:4481–4487. 8223458 10.1002/j.1460-2075.1993.tb06137.x PMC413872

[3] Crompton B. D et al. 2014. The genomic landscape of pediatric Ewing sarcoma. Cancer Discov. 4:1326–1341. 25186949 10.1158/2159-8290.CD-13-1037

[4] Gangwal K et al. 2008. Microsatellites as EWS/FLI response elements in Ewing’s sarcoma. Proc Natl Acad Sci. 105:10149–10154. 18626011 10.1073/pnas.0801073105 PMC2481306

[5] Guillon N et al. 2009. The oncogenic EWS-FLI1 protein binds in vivo GGAA microsatellite sequences with potential transcriptional activation function. PLoS One. 4:e4932. 19305498 10.1371/journal.pone.0004932 PMC2654724

[6] Patel M et al. 2012. Tumor-specific retargeting of an oncogenic transcription factor chimera results in dysregulation of chromatin and transcription. Genome Res. 22:259–270. 22086061 10.1101/gr.125666.111 PMC3266033

[7] Riggi N et al. 2014. EWS-FLI1 utilizes divergent chromatin remodeling mechanisms to directly activate or repress enhancer elements in Ewing sarcoma. Cancer Cell. 26:668–681. 25453903 10.1016/j.ccell.2014.10.004 PMC4492343

[8] Mao X , Miesfeldt S, Yang H, Leiden JM, Thompson CB. 1994. The FLI-1 and chimeric EWS-FLI-1 oncoproteins display similar DNA binding specificities. J Biol Chem. 269:18216–18222. 7517940

[9] Selvanathan SP et al. 2015. Oncogenic fusion protein EWS-FLI1 is a network hub that regulates alternative splicing. Proc Natl Acad Sci. 112:E1307–E1316. 25737553 10.1073/pnas.1500536112 PMC4371969

[10] Grunewald TGP et al. 2018. Ewing sarcoma. Nat Rev Dis Primers. 4:5. 29977059 10.1038/s41572-018-0003-x

[11] Lin PP et al. 2008. EWS-FLI1 induces developmental abnormalities and accelerates sarcoma formation in a transgenic mouse model. Cancer Res. 68:8968–8975. 18974141 10.1158/0008-5472.CAN-08-0573 PMC4167779

[12] Minas TZ et al. 2017. Combined experience of six independent laboratories attempting to create an Ewing sarcoma mouse model. Oncotarget. 8:34141–34163. 27191748 10.18632/oncotarget.9388 PMC5470957

[13] Torchia EC , Boyd K, Rehg JE, Qu C, Baker SJ. 2007. EWS/FLI-1 induces rapid onset of myeloid/erythroid leukemia in mice. Mol Cell Biol. 27:7918–7934. 17875932 10.1128/MCB.00099-07 PMC2169157

[14] Leacock SW et al. 2012. A zebrafish transgenic model of Ewing’s sarcoma reveals conserved mediators of EWS-FLI1 tumorigenesis. Disease Models & Mechanisms. 5:95–106. 21979944 10.1242/dmm.007401 PMC3255547

[15] Vasileva E , Warren M, Triche TJ, Amatruda JF. 2022. Dysregulated heparan sulfate proteoglycan metabolism promotes Ewing sarcoma tumor growth. ELife. 11:e69734. 35285802 10.7554/eLife.69734 PMC8942468

[16] Nielsen SW . 1976. Comparative pathology of bone tumors in animals, with particular emphasis on the dog. Recent Results Cancer Res. 54:3–16. 10.1007/978-3-642-80997-2_2 796921

[17] Sharkey FE , Fogh J. 1979. Incidence and pathological features of spontaneous tumors in athymic nude mice. Cancer Res. 39:833–839. 427772

[18] Hu Y et al. 2011. An integrative approach to ortholog prediction for disease-focused and other functional studies. BMC Bioinf. 12:357. 10.1186/1471-2105-12-357 PMC3179972 21880147

[19] Bailey MH et al. 2018. Comprehensive characterization of cancer driver genes and mutations. Cell. 173:371–385.e18. 29625053 10.1016/j.cell.2018.02.060 PMC6029450

[20] Bellen HJ , Wangler MF, Yamamoto S. 2019. The fruit fly at the interface of diagnosis and pathogenic mechanisms of rare and common human diseases. Hum Mol Genet. 28:R207–R214. 31227826 10.1093/hmg/ddz135 PMC6872428

[21] Sweeney ST , Hidalgo A, de Belle JS, Keshishian H. 2012. Genetic systems for functional cell ablation in Drosophila. Cold Spring Harb Protoc. 2012:950–956. 22949708 10.1101/pdb.top068361

[22] Arvand A , Welford SM, Teitell MA, Denny CT. 2001. The COOH-terminal domain of FLI-1 is necessary for full tumorigenesis and transcriptional modulation by EWS/FLI-1. Cancer Res. 61:5311–5317. 11431376

[23] Boone MA et al. 2021. The FLI portion of EWS/FLI contributes a transcriptional regulatory function that is distinct and separable from its DNA-binding function in Ewing sarcoma. Oncogene. 40:4759–4769. 34145397 10.1038/s41388-021-01876-5 PMC8298202

[24] Smith R et al. 2006. Expression profiling of EWS/FLI identifies NKX2.2 as a critical target gene in Ewing’s sarcoma. Cancer Cell. 9:405–416. 16697960 10.1016/j.ccr.2006.04.004

[25] Luo W et al. 2018. Protein phosphatase 1 regulatory subunit 1A in ewing sarcoma tumorigenesis and metastasis. Oncogene. 37:798–809. 29059150 10.1038/onc.2017.378

[26] Riggi N et al. 2010. EWS-FLI-1 modulates miRNA145 and SOX2 expression to initiate mesenchymal stem cell reprogramming toward Ewing sarcoma cancer stem cells. Genes Dev. 24:916–932. 20382729 10.1101/gad.1899710 PMC2861191

[27] Deneen B , Denny CT. 2001. Loss of p16 pathways stabilizes EWS/FLI1 expression and complements EWS/FLI1 mediated transformation. Oncogene. 20:6731–6741. 11709708 10.1038/sj.onc.1204875

[28] Lessnick SL , Dacwag CS, Golub TR. 2002. The Ewing’s sarcoma oncoprotein EWS/FLI induces a p53-dependent growth arrest in primary human fibroblasts. Cancer Cell. 1:393–401. 12086853 10.1016/s1535-6108(02)00056-9

[29] Azpiazu N , Morata G. 2000. Function and regulation of homothorax in the wing imaginal disc of Drosophila. Development. 127:2685–2693. 10821766 10.1242/dev.127.12.2685

[30] Zykova TY , Levitsky VG, Belyaeva ES, Zhimulev IF. 2018. Polytene chromosomes—a portrait of functional organization of the Drosophila genome. Curr Genomics. 19:179–191. 29606905 10.2174/1389202918666171016123830 PMC5850506

[31] Hu-Lieskovan S et al. 2005. EWS-FLI1 fusion protein up-regulates critical genes in neural crest development and is responsible for the observed phenotype of Ewing’s family of tumors. Cancer Res. 65:4633–4644. 15930281 10.1158/0008-5472.CAN-04-2857

[32] Rorie CJ et al. 2004. The Ews/Fli-1 fusion gene switches the differentiation program of neuroblastomas to Ewing sarcoma/peripheral primitive neuroectodermal tumors. Cancer Res. 64:1266–1277. 14973077 10.1158/0008-5472.can-03-3274

[33] Franzetti GA et al. 2017. Cell-to-cell heterogeneity of EWSR1-FLI1 activity determines proliferation/migration choices in Ewing sarcoma cells. Oncogene. 36:3505–3514. 28135250 10.1038/onc.2016.498 PMC5541267

[34] Staege MS et al. 2004. DNA microarrays reveal relationship of Ewing family tumors to both endothelial and fetal neural crest-derived cells and define novel targets. Cancer Res. 64:8213–8221. 15548687 10.1158/0008-5472.CAN-03-4059

[35] Hancock JD , Lessnick SL. 2008. A transcriptional profiling meta-analysis reveals a core EWS-FLI gene expression signature. Cell Cycle. 7:250–256. 18256529 10.4161/cc.7.2.5229

[36] Kauer M et al. 2009. A molecular function map of Ewing’s sarcoma. PLoS One. 4:e5415. 19404404 10.1371/journal.pone.0005415 PMC2671847

[37] Mootha VK et al. 2003. PGC-1alpha-responsive genes involved in oxidative phosphorylation are coordinately downregulated in human diabetes. Nat Genet. 34:267–273. 12808457 10.1038/ng1180

[38] Subramanian A et al. 2005. Gene set enrichment analysis: a knowledge-based approach for interpreting genome-wide expression profiles. Proc Natl Acad Sci. 102:15545–15550. 16199517 10.1073/pnas.0506580102 PMC1239896

[39] Cironi L et al. 2008. IGF1 is a common target gene of Ewing's sarcoma fusion proteins in mesenchymal progenitor cells. PLoS One. 3:e2634. 18648544 10.1371/journal.pone.0002634 PMC2481291

[40] Petermann R et al. 1998. Oncogenic EWS-Fli1 interacts with hsRPB7, a subunit of human RNA polymerase II. Oncogene. 17:603–610. 9704926 10.1038/sj.onc.1201964

[41] Yang L , Chansky HA, Hickstein DD. 2000. EWS.Fli-1 fusion protein interacts with hyperphosphorylated RNA polymerase II and interferes with serine-arginine protein-mediated RNA splicing. J Biol Chem. 275:37612–37618. 10982800 10.1074/jbc.M005739200

[42] Chong S et al. 2018. Imaging dynamic and selective low-complexity domain interactions that control gene transcription. Science. 361:eaar2555. 29930090 10.1126/science.aar2555 PMC6961784

[43] Lindén M et al. 2019. FET family fusion oncoproteins target the SWI/SNF chromatin remodeling complex. EMBO Rep. 20:e45766. 30962207 10.15252/embr.201845766 PMC6500973

[44] Boulay G et al. 2017. Cancer-specific retargeting of BAF complexes by a prion-like domain. Cell. 171:163–178.e19. 28844694 10.1016/j.cell.2017.07.036 PMC6791823

[45] Sankar S et al. 2013. Mechanism and relevance of EWS/FLI-mediated transcriptional repression in Ewing sarcoma. Oncogene. 32:5089–5100. 23178492 10.1038/onc.2012.525 PMC3899696

[46] Deuring R et al. 2000. The ISWI chromatin-remodeling protein is required for gene expression and the maintenance of higher order chromatin structure in vivo. Mol Cell. 5:355–365. 10882076 10.1016/s1097-2765(00)80430-x

[47] Tsukiyama T , Daniel C, Tamkun J, Wu C. 1995. ISWI, a member of the SWI2/SNF2 ATPase family, encodes the 140 kDa subunit of the nucleosome remodeling factor. Cell. 83:1021–1026. 8521502 10.1016/0092-8674(95)90217-1

[48] Lagarou A et al. 2008. dKDM2 couples histone H2A ubiquitylation to histone H3 demethylation during Polycomb group silencing. Genes Dev. 22:2799–2810. 18923078 10.1101/gad.484208 PMC2569881

[49] Sanchez-Molina S et al. 2020. RING1B recruits EWSR1-FLI1 and cooperates in the remodeling of chromatin necessary for Ewing sarcoma tumorigenesis. Sci Adv. 6:eaba3058. 33097530 10.1126/sciadv.aba3058 PMC7608835

[50] Elzi DJ , Song M, Houghton PJ, Chen Y, Shiio Y. 2015. The role of FLI-1-EWS, a fusion gene reciprocal to EWS-FLI-1, in Ewing sarcoma. Genes Cancer. 6;452–461. 26807198 10.18632/genesandcancer.86 PMC4701224

[51] Fu XD , Ares M Jr. 2014. Context-dependent control of alternative splicing by RNA-binding proteins. Nat Rev Genet. 15:689–701. 25112293 10.1038/nrg3778 PMC4440546

[52] Knoop LL , Baker SJ. 2001. EWS/FLI alters 5’-splice site selection. J Biol Chem. 276:22317–22322. 11301318 10.1074/jbc.M008950200

[53] Knoop LL , Baker SJ. 2000. The splicing factor U1C represses EWS/FLI-mediated transactivation. J Biol Chem. 275:24865–24871. 10827180 10.1074/jbc.M001661200

[54] Grainger RJ , Beggs JD. 2005. Prp8 protein: at the heart of the spliceosome. RNA. 11:533–557. 15840809 10.1261/rna.2220705 PMC1370742

[55] Chanarat S , Strasser K. 2013. Splicing and beyond: the many faces of the Prp19 complex. Biochim Biophys Acta—Mol Cell Res. 1833:2126–2134. 10.1016/j.bbamcr.2013.05.023 23742842

[56] Tapial J et al. 2017. An atlas of alternative splicing profiles and functional associations reveals new regulatory programs and genes that simultaneously express multiple major isoforms. Genome Res. 27:1759–1768. 28855263 10.1101/gr.220962.117 PMC5630039

[57] Torres-Mendez A et al. 2019. A novel protein domain in an ancestral splicing factor drove the evolution of neural microexons. Nat Ecol Evol. 3:691–701. 30833759 10.1038/s41559-019-0813-6

[58] Roy SW , Gilbert W. 2006. The evolution of spliceosomal introns: patterns, puzzles and progress. Nat Rev Genet. 7:211–221. 16485020 10.1038/nrg1807

[59] Chen EY et al. 2013. Enrichr: interactive and collaborative HTML5 gene list enrichment analysis tool. BMC Bioinf. 14:128. 10.1186/1471-2105-14-128 PMC3637064 23586463

[60] Kuleshov MV et al. 2016. Enrichr: a comprehensive gene set enrichment analysis web server 2016 update. Nucleic Acids Res. 44:W90–W97. 27141961 10.1093/nar/gkw377 PMC4987924

[61] Fletcher JC , Thummel CS. 1995. The ecdysone-inducible broad-complex and E74 early genes interact to regulate target gene transcription and Drosophila metamorphosis. Genetics. 141:1025–1035. 8582609 10.1093/genetics/141.3.1025 PMC1206826

[62] Urness LD , Thummel CS. 1995. Molecular analysis of a steroid-induced regulatory hierarchy: the Drosophila E74A protein directly regulates L71-6 transcription. EMBO J. 14:6239–6246. 8557043 10.1002/j.1460-2075.1995.tb00314.x PMC394748

[63] Monument MJ et al. 2014. Clinical and biochemical function of polymorphic NR0B1 GGAA-microsatellites in Ewing sarcoma: a report from the Children’s Oncology Group. PLoS One. 9:e104378. 25093581 10.1371/journal.pone.0104378 PMC4122435

[64] Johnson KM et al. 2017. Role for the EWS domain of EWS/FLI in binding GGAA-microsatellites required for Ewing sarcoma anchorage independent growth. Proc Natl Acad Sci. 114:9870–9875. 28847958 10.1073/pnas.1701872114 PMC5603999

