# Supplementary material for: Human EWS-FLI protein recapitulates in Drosophila the neomorphic functions that induce Ewing sarcoma tumorigenesis
Source: PNAS Nexus. 2022 Oct 6;1(4):pgac222. doi: 10.1093/pnasnexus/pgac222 (PMC9802468; doi:10.1093/pnasnexus/pgac222)
Supplement: pgac222_Supplemental_Files [file pgac222_supplemental_files.zip › PNASNEXUS-PNASNEXUS-2022-00391-T-s02.docx]

**
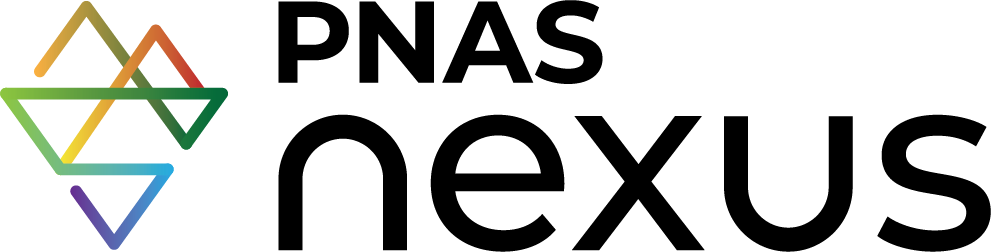
**

**Supplementary Information for**

**Human EWS-FLI protein recapitulates in Drosophila the neomorphic functions that induce Ewing sarcoma tumorigenesis**.

Cristina Molnar, Jose Reina, Anastasia Herrero, Jan Peter Heinen, Victoria Méndiz, Sophie Bonnal, Manuel Irimia, María Sánchez-Jiménez, Sara Sánchez-Molina, Jaume Mora and Cayetano Gonzalez.

Corresponding authors: Cayetano Gonzalez and Jaume Mora.

Email: [gonzalez@irbbarcelona.org](mailto:gonzalez@irbbarcelona.org) and [jaume.mora@sjd.es](mailto:jaume.mora@sjd.es)

**This PDF file includes:**

Materials and Methods

Figures S1 to S9

Table S1 to S3

SI References

**MATERIALS AND METHODS**

**Fly stocks**

The following fly strains were used in this study: *nub-Gal4* (65) and *sal^EPv^-Gal4* (66), and *UAS-EWS-FLI_1FS_*, *UAS-EWS-FLI_2FS_*, *UAS-CazEts65a*, *20X(GGAA)µSat-YFP*, *10X(GGAA)µSat-YFP*, *3X(GGAA)µSat-YFP* and *1X(GGAA)µSat-YFP* (this work). The strains *w; UAS-EWS-FLI_1FS_ 20X(GGAA)µSat-YFP/CyO, w; sal^EPv^-Gal4 UAS- UAS-EWS-FLI_1FS_ 20X(GGAA)µSat-YFP/CyO, w; UAS-EWS-FLI_2FS_ 20X(GGAA)µSat-YFP/CyO* and *w; UAS-Caz-Ets65a 20X(GGAA)µSat-YFP/CyO* were made by 2R chromosome recombination. The TRiP RNAi stocks from Bloomington Drosophila Stock Center (BDSC) used are specific for targeting the following genes: *Bap111* (#26218), *Bap170* (#26308), *Bap55* (#31708), *Bap60* (#33954), *brm* (#34520), *e(y)3* (#32346), *mor* (#34919), *osa* (#38285), *polybromo* (#32840), and *Snr1* (#32372). All the Gal4 lines in Table S1 are described in Flybase. The wild type strain used was *w^1118^*. All crosses, including controls, were maintained at 25ºC.

**Genotypes and crossing schemes**

For all the transcriptomics and proteomics experiments, females *yw; nub-Gal4* were crossed to males *w^1118^* and to males *w; UAS-EWS-FLI_1FS_/CyO,* to obtain *nub-Gal4/+* (control) and *nub-Gal4/UAS-EWS-FLI_1FS_* (EWSFLI) wandering third instar male larvae. For a second microarray expression profiling experiment, females *w*; *sal^EPv^-Gal4* and females *w*; *sal^EPv^-Gal4 UAS-EWS-FLI_1FS_ /CyO* were crossed to males *w^1118^* and to males *mor^RNAi^,* to obtain *sal^EPv^-Gal4/+* (control), *sal^EPv^-Gal4/mor^RNAi^* (mor)*_,_ sal^EPv^-Gal4 UAS-EWS-FLI_1FS_ /+* (EWSFLI) and *sal^EPv^-Gal4 UAS-EWS-FLI_1FS_ /mor^RNAi^* (EWSFLI_mor) wandering third instar male larvae.

For quantified GGAAµSat>YFP expression, 10 females *w; sal^EPv^-Gal4 UAS-EWS-FLI_1FS_ /CyO* were crossed to 5 males carrying each of the transgenic 1X, 3X, 10X and 20X(GGAA)µSat constructs. For quantified 20X(GGAA)µSat>GFP expression in depleted condition for the components of BAP/PBAP complex, 10 females *w; sal^EPv^-Gal4 UAS-EWS-FLI_1FS_ 20X(GGAA)µSat-YFP/CyO* were crossed to 5 males carrying each of the TRiP RNAi lines listed above. Wandering third instar larvae from each cross were dissected, fixed and mounted to visualized the nX(*GGAA)µSat-YFP* expression in the salivary glands under confocal microscope. All the experiments were performed blind. All individuals analyzed were between 4 and 6 days old.

**Allograft assays**

Wing imaginal discs from wandering third instar larvae were implanted in female hosts of 3-4 days old as described in (67). Implanted hosts were kept at 25ºC.

**Immunohistochemistry**

Immunostaining of salivary glands was performed as follows: salivary glands were dissected in phosphate-buffered saline (PBS), fixed for 30 min in 4% formaldehyde with 0.3% TritonX-100, washed 3 times in PBS-0.3% Triton X-100 (PBST) and blocked for 90 min in PBST with 10% fetal calf serum (PBSTF). Primary and secondary antibodies were incubated in PBSTF overnight at 4ºC. Primary antibodies used in this study include anti-EWS rabbit Affinity Purified (Bethyl Cat# A300-417A). We used Alexa Fluor secondary antibodies (1:1000, Life Technologies). DNA was stained with DAPI. Salivary glands were mounted in Vectashield (Molecular Probes). Images were acquired with a SP8 Leica confocal image microscope and processed in Adobe Photoshop CS6 and ImageJ.

**Quantification and Statistical Analysis**

Quantification of YFP fluorescence levels was carried out using ImageJ to calculate the mean grey values of each Region Of Interest (ROI) in a focal plane per salivary gland acquired with an SP8 Leica confocal image microscope. The results were represented in boxplots, and *P* values were calculated by unpaired *t* test using GraphPad Prism 9.2 for MacOS X (GraphPad Software, La Jolla, CA, USA) ([www.graphpad.com](http://www.graphpad.com)).

**Cloning and transgenics**

Constructs p20X(GGAA)µSat-YFP, p10X(GGAA)µSat-YFP, p3X(GGAA)µSat-YFP and p1X(GGAA)µSat-YFP were made using *pUASt-attB-YFP* (68) as backbone, and UAS sequences were replaced by a fragment containing 20, 10, 3 and 1 repetitions of GGAA, respectively, followed by a minimal heat-shock promoter (HSP) sequence. 20XGGAA fragment was synthetized by GeneWiz. 10XGGAA, 3XGGAA and 1XGGAA fragments were made fusing a dsDNA oligo (Eurofins) that contain 10, 3 and 1 GGAA repeats, respectively, to a PCR fragment containing the minimal HSP. All fragments were cloned into *HindIII* and *EcoRI* sites of the *pUASt-attB-YFP* vector using InFusion (Takara).

Sequences codon optimized for *Drosophila melanogaster* corresponding to the proteins EWS-FLI1 (Q9BZD1), EWS-FLI2 (F1JVV8), FLI1 (QO1543), EWS-ERG (69), EWS-FEV (70), FUS-ERG (71), and Caz-Ets65A were synthetized by GeneWiz. Caz-Ets65A corresponds to the fusion of first 120 amino acids of Caz with the amino acids 259-490 of Ets65A. pUASt-EWS-FLI_1∆69C_ (aa 1-407), pUASt-EWS-FLI_2∆69C_ (aa 1-457), pUASt-EWS-FEV_∆73C_ (aa 1-495), pUASt-EWS-ERG_∆67C_ (aa 1-428), pUASt-FUS-ERG_∆67C_ (aa 1-373), pUASt-EWS-FLI_1FS_, pUASt-EWS-FLI_2FS_ were made by PCR amplification using InFusion (Takara). pUASt-EWS-FEV_FS,_ pUASt-EWS-ERG_FS_ and pUASt-FUS-ERG_FS_ were made introducing gBlocks (IDT) with the corresponding frame shift sequence into the *BsrGI* and *XhoI* restriction sites. All the ORF were cloned in the *pUASt-attB* vector. All transgenics fly lines were generated by Bestgene Inc. All primers used are shown in Table S1.

*Vectors for transfection into human cell lines*

The construct [pCDH-puro-EWS-FLI1](https://www.addgene.org/102813/) full length was obtained from Addgene (#102813). pCDH-puro-EWS-FLI_1FS_ was made introducing gBlocks (IDT) with the frame shift sequence into the *BsrGI* and *XhoI* restriction sites. Control plasmid pCDH-puro-GFP was made inserting the EGFP *NheI-NotI* fragment from pEGFP-N1 in the pCDH vector.

**Transcriptomics**

*Microarray processing*

Dissected Drosophila salivary glands were collected in 45 µl of a lysis buffer containing 20 mM DTT, 10 mM Tris-HCl pH 7.4, 0.5% SDS, and 0.5µg/µl proteinase K, incubated at 65 ºC for 15 minutes and immediately frozen until processing. RNA extraction and cDNA generation was done at the IRB Barcelona Functional Genomics Core Facility. Briefly, RNA was treated with DNAse I and purified using magnetic beads (RNAClean XP, Beckman Coulter). RNA was quantitated with Qubit RNA HS Assay kit (Invitrogen), and RNA integrity was assessed with the Bioanalyzer 2100 RNA Pico assay (Agilent). 25 ng of RNA were reverse transcribed and amplified using the Whole Transcriptome Amplification method (WTA2, Sigma Aldrich) with 17 cycles of amplification. cDNA was further purified using a spin column (PureLink Quick PCR Purification Kit, Invitrogen) and quantified using a microvolume spectrophotometer (Nanodrop ND-1000, Thermo-Fisher Scientific).

For microarray processing, 8 µg of cDNA were fragmented and labelled according to the manufacturer instructions (GeneChip Mapping 250K Nsp Assay Kit, Affymetrix). Array hybridization was performed using the GeneChip Hybridization, Wash, and Stain Kit (Applied Biosystems). Briefly, libraries were denatured at 99°C for 2 min prior to incubation into the Drosophila Genome 2.0 arrays (Applied Biosystems). Libraries were hybridized on the arrays for 16 h at 45 °C for 60 rpm at GeneChip Hybridization Oven 645 (Affymetrix/ThermoFisher Scientific). Washing and Stain steps were performed using a GeneChip Fluidics Station 450 following the Drosophila Genome 2.0 protocol (Affymetrix/ThermoFisher Scientific). Finally, arrays were scanned with a GeneChip Scanner GCS3000 (Affymetrix/ThermoFisher Scientific). The CEL files containing the microarray data were generated with the Command Console software (Affymetrix/ThermoFisher Scientific), and were used for probeset-based gene expression measurements using robust multichip average (RMA) normalization. Results were analyzed using the Transcriptome Analysis Console 4.0 (TAC) software. Genes with an absolute FC of >2 and a FDR p-value of <0.05 were considered differentially expressed.

*Quantitative real-time PCR*

Dissected Drosophila larval salivary glands were collected in 45 µl of a lysis buffer containing 20 mM DTT, 10 mM Tris-HCl pH 7.4, 0.5% SDS, and 0.5µg/µl proteinase K, incubated at 65 ºC for 15 minutes and immediately frozen until processing. RNA extraction and cDNA generation was done at the IRB Barcelona Functional Genomics Core Facility. Briefly, RNA was treated with DNAse I and purified using magnetic beads (RNAClean XP, Beckman Coulter). RNA was quantitated with Qubit RNA HS Assay kit (Invitrogen), and integrity assessed with the Bioanalyzer 2100 RNA Nano assay (Agilent). 25 ng of RNA was reverse transcribed and amplified using the Whole Transcriptome Amplification method (WTA2, Sigma Aldrich) with 17 cycles of amplification. cDNA was further purified using a spin column (PureLink Quick PCR Purification Kit, Invitrogen) and quantified using a microvolume spectrophotometer (Nanodrop ND-1000, Thermo-Fisher Scientific). cDNA yield ranged from 7.3 to 13.2 µg.

PowerUp SYBR Green Master Mix (Thermo Fisher Scientific) was used for quantitative real-time PCR following manufacturer’s instructions. The real time assays were conducted in a QuantStudio 6 Flex Real-Time PCR System (Thermo Fisher Scientific) using SYBR Green as the detection system and ROX as reference dye. The primers were designed using Primer-BLAST (NIH). mRNA levels were assessed from three independent RNA extractions and three technical replicates were performed on each sample. For the validation of Affymetrix data experiments RNA levels were normalized to *RpL32*. For quantitative real-time PCR’s from transfected HEK293 cells mRNA levels were normalized to *GADPH* and *HPRT1* housekeeping genes. Only primers with efficiency 90% and 110% were used. The primers employed are shown in Table S1.

*Gene Set Enrichment Analysis (GSEA)*

The GSEA preRanked algorithm was used to compare the human EWS-FLI1 signatures from (32, 33) to all genes in the Drosophila microarrays ranked by average log2FC. Genesets with a FDR q-value of <0.25 were accepted as a significant enrichment. Drosophila orthologs of these human signatures were identified using the DIOPT ortholog mapping online resource (18).

*RNA-seq library preparation and sequencing*

Dissected Drosophila male larval salivary glands were collected in 45 µl of a lysis buffer containing 20 mM DTT, 10 mM Tris-HCl pH 7.4, 0.5% SDS, and 0.5µg/µl proteinase K, incubated at 65 ºC for 15 minutes, and immediately frozen until processing. RNA isolation and library preparation were done at the IRB Barcelona Functional Genomics Core Facility. RNA was treated with DNAse I and purified using magnetic beads (RNAClean XP, Beckman Coulter). RNA was quantitated with Qubit RNA HS Assay kit (Invitrogen), and integrity was assessed with the Bioanalyzer 2100 RNA Nano assay (Agilent). Poly-A mRNA was purified from 660 ng of total RNA using the kit NEBNext Poly(A) mRNA Magnetic Isolation Module (New England Biolabs). Dual-indexed cDNA libraries were generated using the NEBNext Ultra II Directional RNA Library Prep Kit for Illumina (New England Biolabs). Ten minutes of RNA fragmentation and ten cycles of PCR amplification were applied to all libraries. The final libraries were quantified using the Qubit dsDNA HS assay (Invitrogen) and quality controlled with the Bioanalyzer 2100 DNA HS assay (Agilent). An average size of 420 bp was confirmed. An equimolar pool was prepared with the six libraries and submitted for sequencing at the Centre Nacional d'Anàlisi Genòmica (CRG-CNAG). A final quality control by qPCR was performed by the sequencing provider before paired-end 150 nt sequencing on a NovaSeq6000 S4 (Illumina). The sequencing results exceeded 168 Gbp with a minimum of 82 million paired-end reads sequenced for each sample.

*Splicing Analysis*

The toolset *vast-tools v2.5.1* (53) (dm6, VASTDB library: vastdb.dme.23.06.20.tar.gz (54)) was used to quantify alternative splicing from RNA sequencing data obtained from salivary glands control and EWS-FLI_1FS_. Inclusion levels of the alternative sequence were estimated using the ‘percent spliced in’ (PSI) metric for different types of alternative splicing events including alternative exons, intron retention, alternative 3’ and alternative 5’ splice sites. To identify differentially spliced events between control and EWS-FLI_1FS_-expressing samples (three replicates for each condition), we used the *vast-tools compare* module using default parameters. For each tested event, these require all the replicates to have sufficient read coverage to confidently estimate a PSI value (coverage score of VLOW or higher), a minimum average |ΔPSI| of 15 15 between the two groups (control and EWS-FLI_1FS_-expressing samples), and a minimum ΔPSI of 5 between the largest and lowest PSI in each group (further details can be found in https://github.com/vastgroup/vast-tools). Primers for RT-PCR validation assays were selected from VastDB ([vastdb.crg.eu](http://vastdb.crg.eu)). PCR reactions were carried out using DreamTaq enzyme (Thermo) with 40 ng of cDNA. To quantify inclusion of alternatively spliced variants, the PCR products were loaded in a Chip DNA 1000 and run in a Bioanalyzer 2100 (Agilent). The nanomolar content of each band was extracted with 2100 Expert software (Agilent) and PSI values were calculated as the ratio between the inclusion amplicon and the sum of inclusion and skipping amplicons.

BAM files have been generated on the merged triplicates for Control and EWS-FLI conditions using STAR-2.7.1a and dm6 genome annotation, indexed with *samtools*. Sashimi plots have been prepared using *ggsashimi* (<https://github.com/guigolab/ggsashimi>).

**Interactome**

*Immunoprecipitation*

Immunoprecipitation were performed as previously described in (41) with slightly modifications: salivary glands were resuspended in 1m Immunoprecipitation Buffer (IP) (50 mM Tris-HCl pH 8, 150 mM NaCl, 5 mM EDTA, 0.5% IGEPAL 630 and 10% glycerol supplemented with protease and phosphatase inhibitors (Roche) and 1mM Pefabloc (Roche). Resuspended glands were homogenized in a dounce grinder and the homogenized was sonicated in a Branson 550 using micro tip for 3 min (10s On 30s Off) in ice water. Protein supernatant was then collected after centrifugation for 15 min at 14000 rpm and 4°C. The volume of the protein supernatant was raised to 2 ml using IP buffer and clarified by incubation with 30 μl of Dynabeads protein A (Thermo) during 2h at 4ºC. 2μg of antibody anti-EWS (A300-417 Bethyl) and 2 μg of control IgG (rabbit IgG, 12-370 Millipore) were coupled to Dynabeads protein A for 2h in Citrate-Phosphate buffer (24mM citric acid, 51.7 mM Na_2_HPO_4_, pH 5.0) at RT in a multi-rotator, after that, beads were collected with magnetic rack and washed 3 times with IP buffer. The protein supernatant was supplemented with 100 μg/mL ethidium bromide (SIGMA-ALDRICH) and the same volume of protein supernatant was added to the magnetics beads with the antibody anti-EWS and to the IgG control. The sample were incubated with the beads overnight at 4°C in a multi-rotator. Beads were collected with the magnet and washed 5 times with IP buffer, 1 time with 50 mM Tris-HCl pH 8, 250 mM NaCl, 5 mM EDTA, 1 time with 50 mM Tris-HCl pH 8, 500 mM NaCl, 5 mM EDTA and 1 time with 50 mM Ammonium Bicarbonate and resuspended in 50 µl of 50 mM Ammonium Bicarbonate.

*Mass Spectrometry*

Samples were tryptic digested directly on the beads. Digestion was performed with 2 µg trypsin in 50mM NH_4_HCO_3_ at 37°C overnight. Additional 1 µg trypsin were added and incubated 2 more hours at 37°C. The digestion was stopped by adding formic acid to 1% final concentration. Samples were cleaned up through C18 tips (polyLC C18 tips) and peptides were eluted with 80% acetonitrile/1% TFA. Finally, samples were evaporated to dryness and reconstituted in 50 µL 3% acetonitrile/1% formic acid aqueous solution and diluted 1/8 for MS analysis. Samples were processed in a Orbitrap Fusion Lumos™ Tribrid. The nano-LC-MS/MS set up was as follows. Digested peptides were diluted in 3% ACN/1% FA. Sample was loaded to a 300 µm × 5 mm PepMap100, 5 µm, 100 Å, C18 µ-precolumn (Thermo Scientific) at a flow rate of 15 µl/min using a Thermo Scientific Dionex Ultimate 3000 chromatographic system (Thermo Scientific). Peptides were separated using a C18 analytical column NanoEase MZ HSS T3 column (75 μm × 250 mm, 1.8 μm, 100A) (Waters) with a 90 min run, comprising three consecutive steps with linear gradients from 3 to 35% B in 60 min, from 35 to 50% B in 5 min, and from 50 % to 85 % B in 2 min, followed by isocratic elution at 85 % B in 5 min and stabilization to initial conditions (A= 0.1% FA in water, B= 0.1% FA in CH3CN). The column outlet was directly connected to an Advion TriVersa NanoMate (Advion) fitted on an Orbitrap Fusion Lumos™ Tribrid (Thermo Scientific). The mass spectrometer was operated in a data-dependent acquisition (DDA) mode. Survey MS scans were acquired in the Orbitrap with the resolution (defined at 200 m/z) set to 120,000. The lock mass was user-defined at 445.12 m/z in each Orbitrap scan. The top speed (most intense) ions per scan were fragmented by CID and detected in the linear ion trap. The ion count target value was 400,000 and 10,000 for the survey scan and for the MS/MS scan respectively. Target ions already selected for MS/MS were dynamically excluded for 15s. Spray voltage in the NanoMate source was set to 1.60 kV. RF Lens were tuned to 30%. Minimal signal required to trigger MS to MS/MS switch was set to 5,000. The spectrometer was working in positive polarity mode and singly charge state precursors were rejected for fragmentation.

*Database search*

We performed a twin database search with two separated softwares, Thermo Proteome Discoverer v2.5.0.400 (PD) and MaxQuant v1.6.17.0 (MQ). The search engine nodes used were Sequest HT for PD and Andromeda for MQ. The databases used in the search was UniProt DROME (released 2021_02), a database of the user protein EWS Human and contaminants. We run the search against targeted and decoy databases to determine the false discovery rate (FDR). Search parameters included trypsin enzyme specificity, allowing for two missed cleavage sites, oxidation in M and acetylation in protein N-terminus as dynamic modifications. Peptide mass tolerance was 10 ppm and the MS/MS tolerance was 0.6 Da. Peptides with a q-value lower than 0.01 and a FDR < 1% were considered as positive identifications with a high confidence level.

*Quantitative Analysis*

For the quantitative analysis, contaminant identifications were removed and unique peptide spectrum matches of protein groups identified with Sequest HT and Andromeda were analyzed with SAINTexpress-spc v3.11 (SAINTe). SAINTe compares the prey control spectral counts with the prey test spectral counts for all available technical replicates. For each available bait and for each available replicate, we took as prey count the maximum count result between PD and MQ. Once obtained this combined dataset, we ran the SAINTe algorithm. High confidence interactors were defined as those with Bayesian false discovery rate BFDR ≤ 0.02 and fold change FC ≥ 3.

**GO and KEGG analysis**

Functional annotation of GO terms and KEGG pathways was performed using the online tool Database for Annotation, Visualization and Integrated Discovery (DAVID 6.8; http://david.abcc.ncifcrf.gov/). Terms with a *p*-value of <0.05 were accepted as a significant enrichment.

**Cell culture, lentivirus infection, and transfection**

The human embryonic kidney HEK293 and HEK293T containing AgT from SV40, were cultured in RPMI-1640 media (Gibco) and supplemented with 10% fetal bovine serum, l-glutamine, and penicillin/streptomycin. Cells were cultured at 37°C with 5% CO2.

Empty pCDH, pCDH-EWS-FLI full length, and pCDH -EWS-FLI_1FS_ expressing lentiviruses were produced in Lenti-X 293T packaging cells (Takara, Cultek) at a low passage number. For each plate, 4 μg of the lentiviral plasmid, 4 μg of the envelope plasmid (VSV-G) and 4 μg of the packaging plasmids (RRE and RSV) were introduced by PEI transfection (Polyethylenimine, Sigma Aldrich), according to standard protocols. The supernatant containing lentiviruses was collected 24 hours after transfection. The HEK293T cell lines were seeded at 3x10^5 cells/well in 6-well plates and transduced with 1:1 of the lentiviral supernatant with fresh media containing Polybrene (Sigma Aldrich) at 1 µg/mL. Cells were selected with fresh growth media containing puromycin (2 μg/mL) for 72 hours. A control dish without the transduction media was also selected with puromycin, to control for killing of non-transduced cells.

HEK293 cells were transfected using Lipofectamine 3000. Cells were seeded on a 6-well plate at a density to ensure 70-80% confluent cultures at 24 hr after seeding. Transfection was performed using 2µg of DNA mixed with 250µl OptiMEM medium (Thermo Fisher Scientific; #31985062), 5µl Lipofectamine 3000 Reagent and 5µl P3000 reagent (Life Technologies; #L3000001). The DNA/Lipofectamine solution was incubated at room temperature for 15 min. After incubation, the DNA/Lipofectamine mix was added to cells.

**Clonogenic assays**

HEK293T cell (750 cells/well) infected with empty vector, full-length EWS-FLI or EWS-FLI_1FS_ expressing lentiviruses were seeded in 6-well plates and media was changed every 2-3 days until visible colonies were grown. Cells were fixed with 4% formaldehyde for 30 minutes, washed with Dulbeco’s PBS (DPBS), stained with a crystal violet solution (2% W/V, 20% methanol in PBS) for 5 minutes and washed with water. Colony area was quantified using ImageJ.


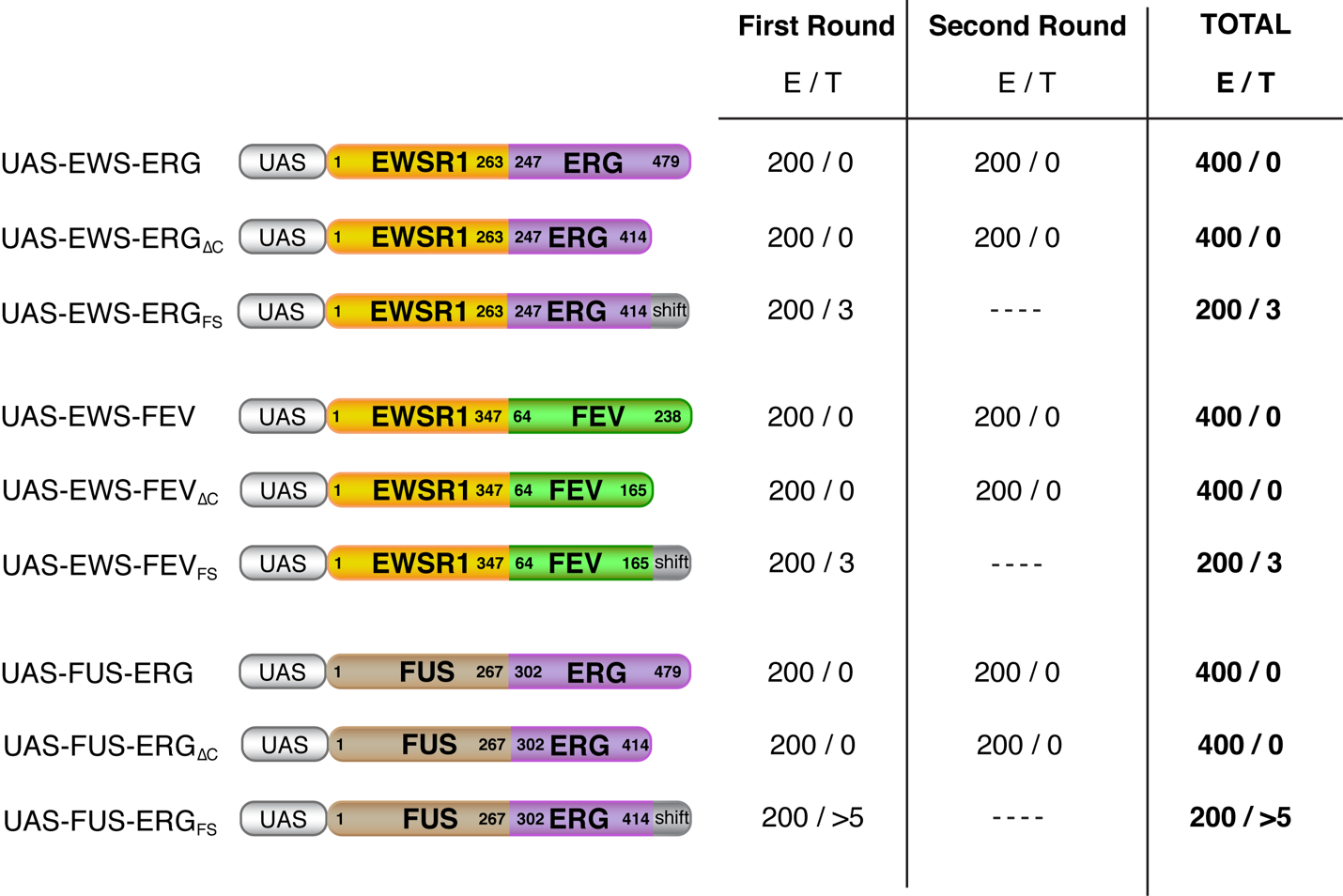


Fig. S1. UAS transgenes encoding human EWSR1-ERG, EWSR1-FEV, and FUS-ERG oncogenes are highly toxic in Drosophila.

Number of injected embryos “E” and transgenic animals recovered “T” in two rounds of injection using the ZH-51C landing site. UAS transgenes carrying the full length, or the equivalent C-terminal deletion or frameshift versions of the human EWS-ERG, EWS-FEV, and FUS-ERG fusion proteins.


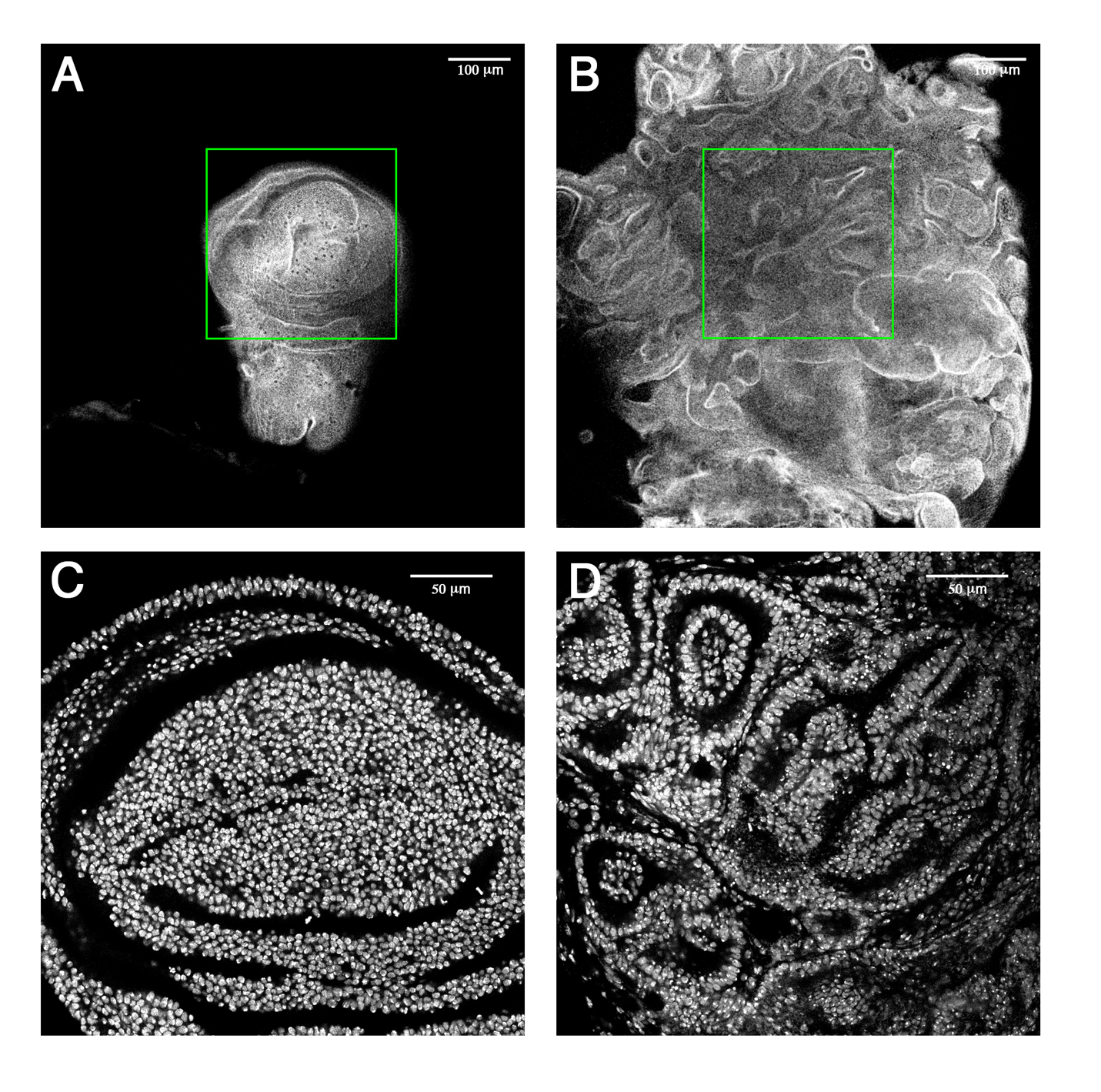


Fig. S2. EWS-FLI1FS induces epithelial malignant tumors in the developing wing discs.

Wing imaginal disc from a wild-type third instar larvae **(A, C)** and wing disc tumor after implantation in an adult host **(B, D)**. Green squares in A and B represent the area shown in C and D. Scale bar=100 µm in A and B. Scale bar=50 µm in C and D.


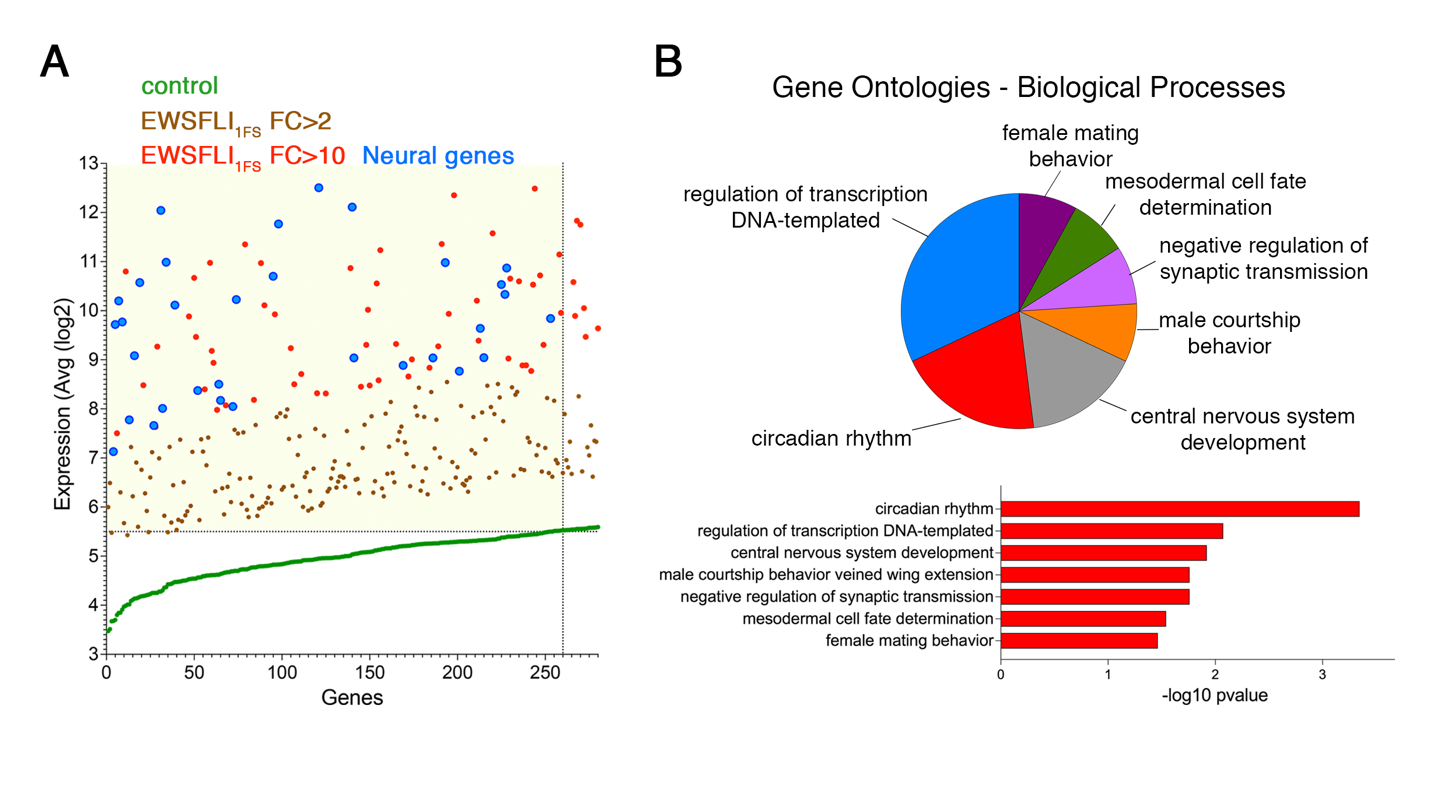


Fig. S3. EWS-FLI1FS signature includes genes associated with neural development.

**(A)** Expression levels of the EWS-FLI1FS upregulated genes with little or no expression in control salivary glands, in control (green; *nub-Gal4/+*) and EWS-FLI1FS (brown, red and blue; *nub-Gal4/UAS-EWS-FLI1FS*) samples. A subset of 86 genes are upregulated with FC>10 (red), and includes 32 neural genes (blue). The genes are ordered along the *x-*axis as a function of their expression levels in control samples. **(B)** Gene Ontology Biological Processes terms significantly enriched in the set of 86 genes highly upregulated (FC>10) (*p*-values < 0.05). Pie and Bar graphs represent the percentage of genes and the *p*-values (-log10) of each term, respectively.


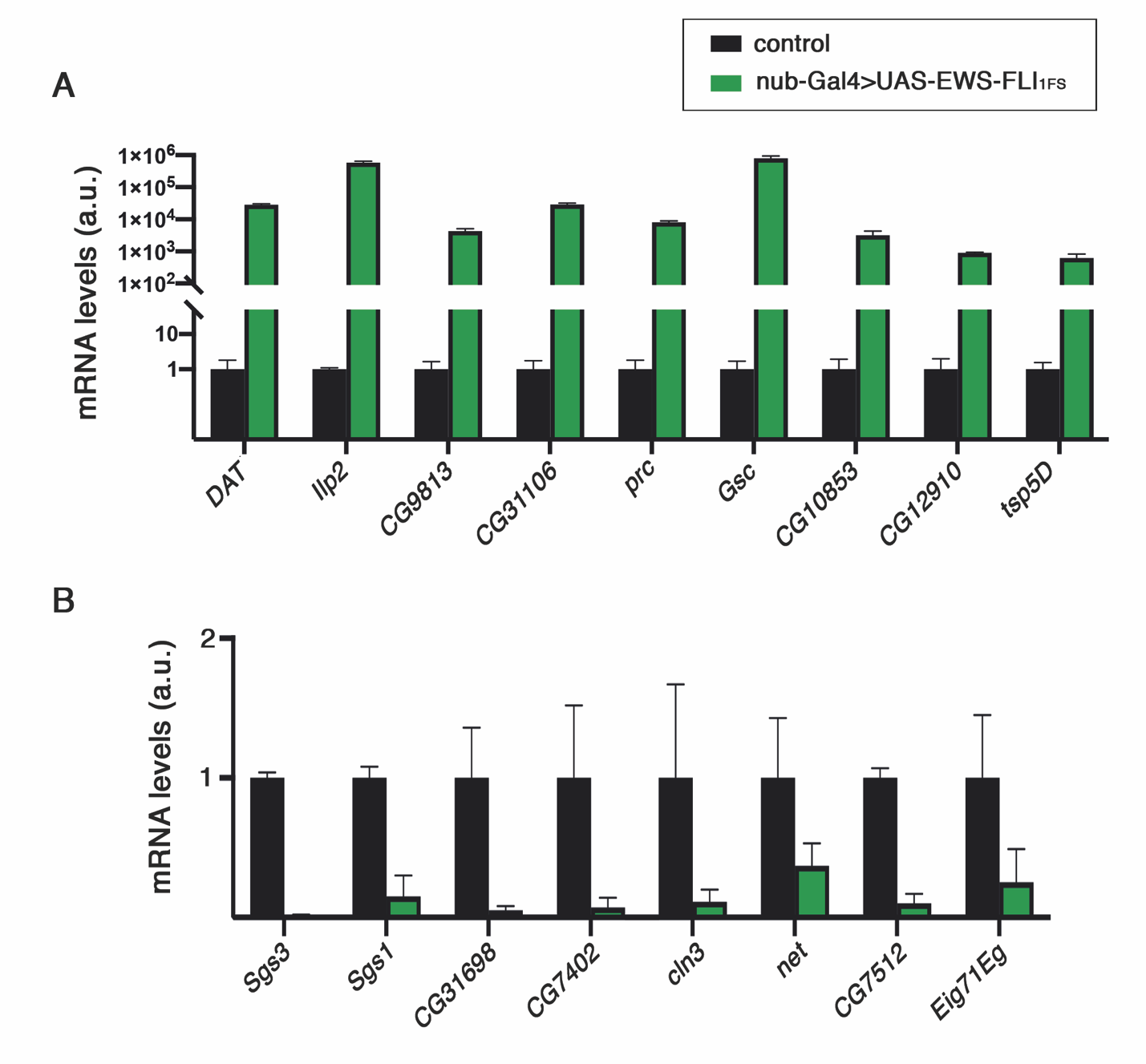


Fig. S4. Validation of Affymetrix data by quantitative real-time PCR.

Quantification by RT-qPCR of **(A)** upregulated and **(B)** downregulated genes in EWS-FLI1FS (green; *nub-Gal4/UAS-EWS-FLI1FS* compared to control (black; *nub-Gal4/+*) salivary glands. Error bars indicate SEM of data derived from three biological replicates (two technical duplicates each). a.u., arbitrary units.


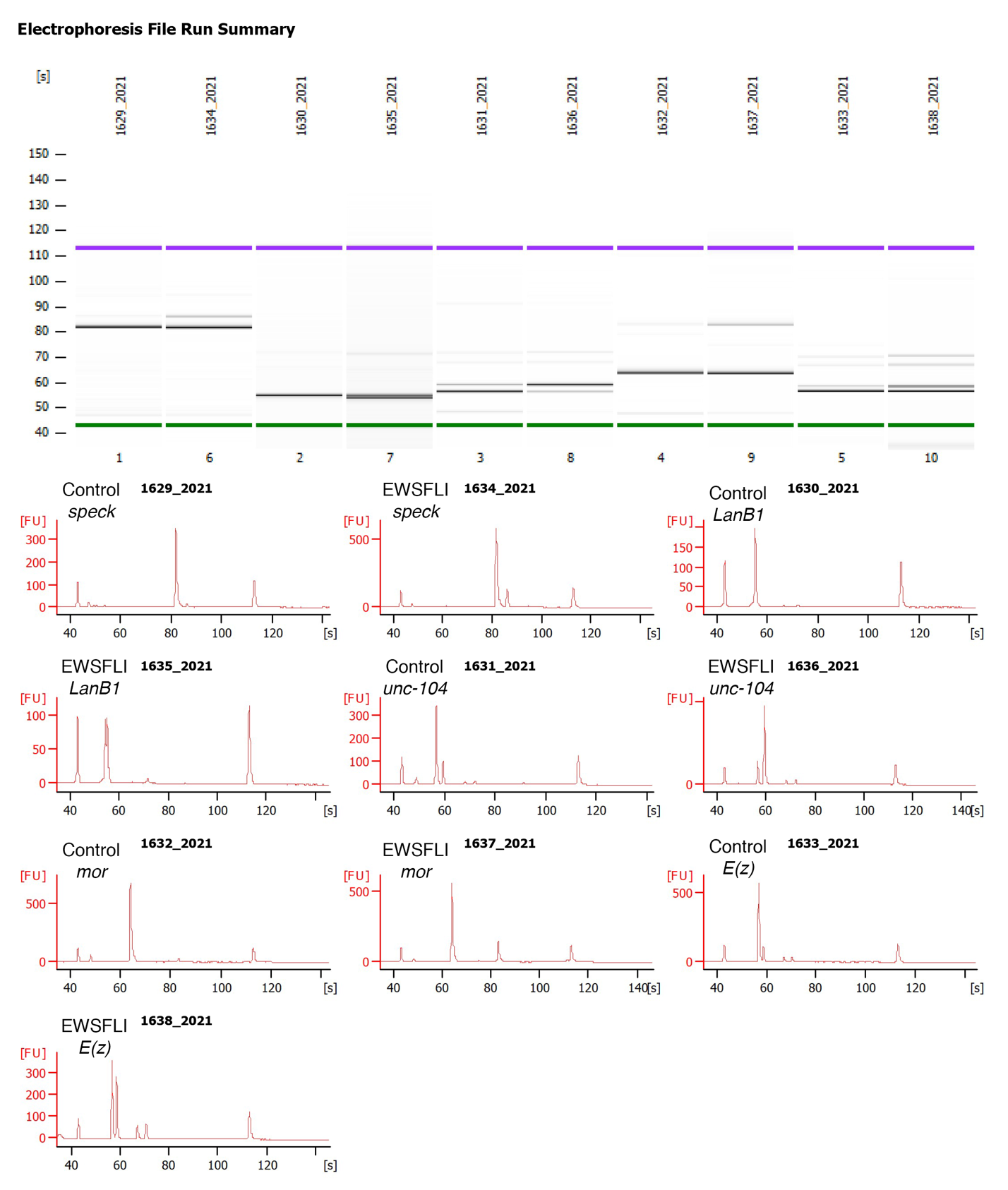


Fig. S5. Validation of selected alternative splicing events by RT-PCR.

Electrophoresis File Run Summary from the Bioanalyser 2000, showing the gel image and the electropherograms of each sample.


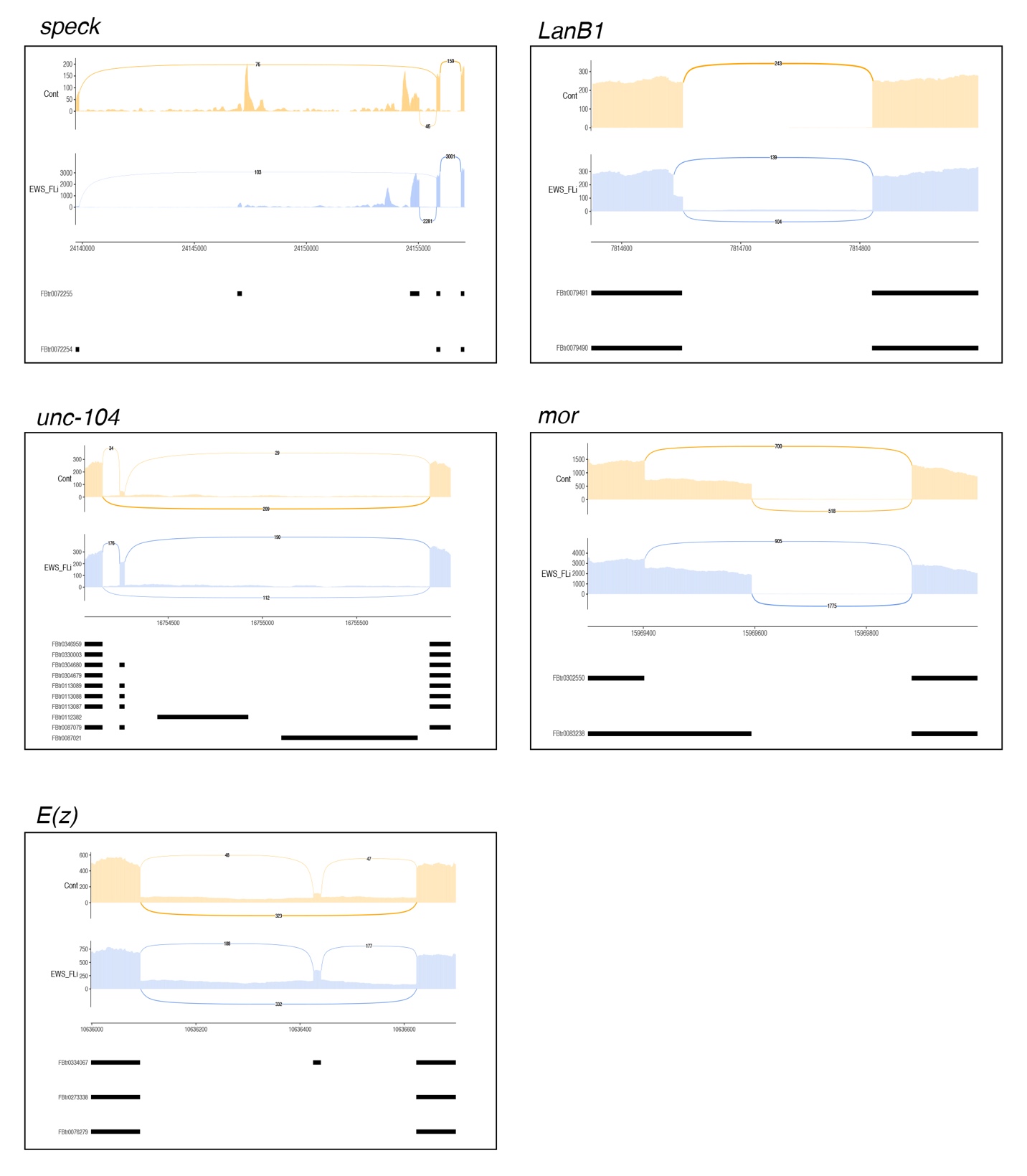


**Fig. S6.** **Sashimi plots of selected alternative splicing events.**

Sashimi plots of five genes selected for validation by RT-PCR.


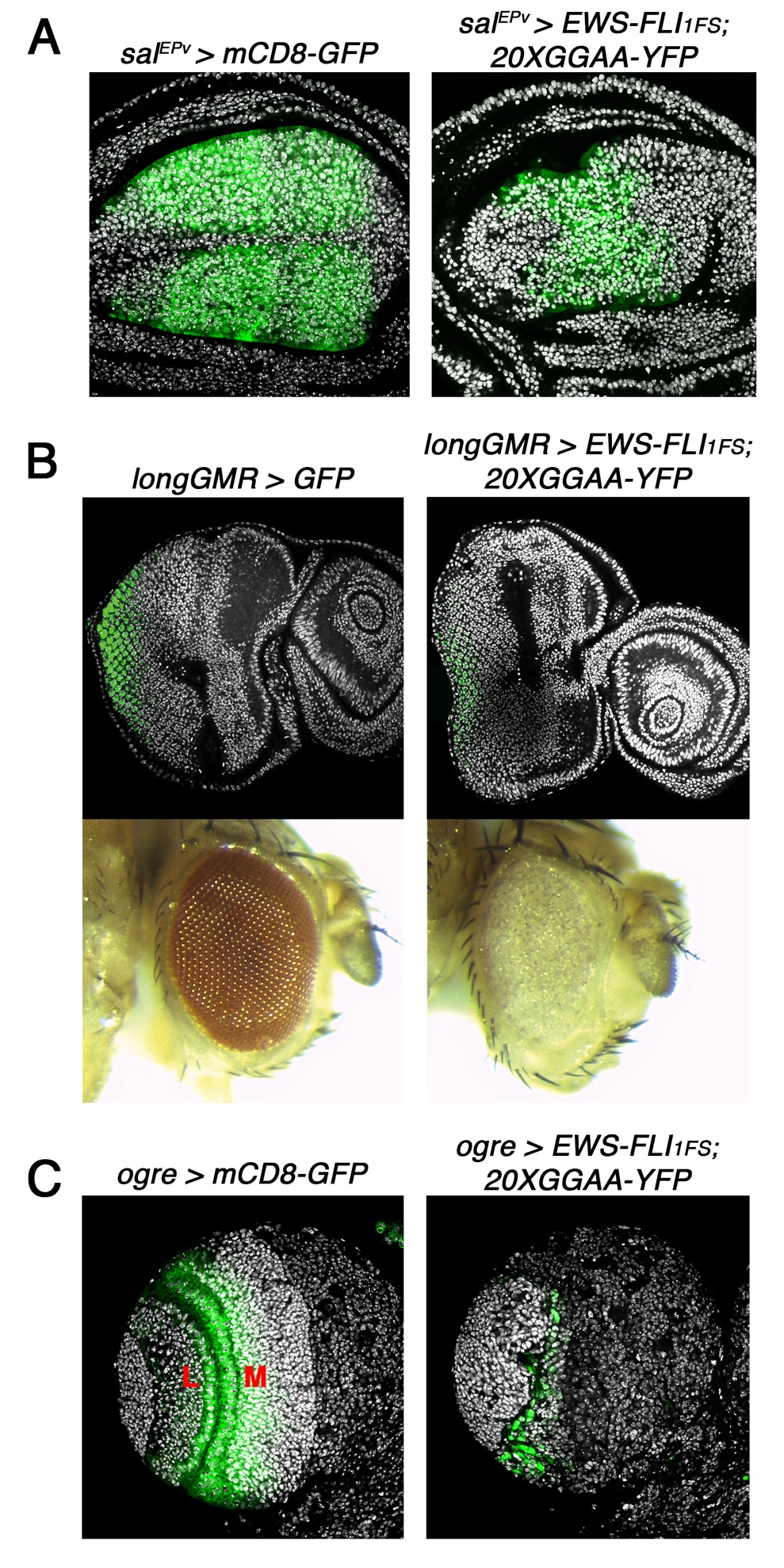


**Fig. S7.** **Activation of 20X(GGAA)µSat-YFP by EWS-FLI1FS larval discs and brains.**

(**A**) Wing imaginal discs from larvae expressing mCD8-GFP or EWS-FLI_1FS_ in the wing pouch with *sal^EPv^-Gal4.* (**B**) Eye imaginal discs from larvae expressing GFP or EWS-FLI_1FS_ in the ommatidia with *longGMR-Gal4*. The resulting adult eyes from longGMR>EWS-FLI_1FS_ are strongly rough and present a nearly total loss of eye pigmentation. (**C**) Larval brain lobes from larvae expressing mCD8-GFP or EWS-FLI_1FS_ in the neuroepithelium (NE) with *ogre-Gal4*. EWS-FLI_1FS_ expression leads to loss of most of the NE and NE-derived medulla. L=lamina, M=medulla.


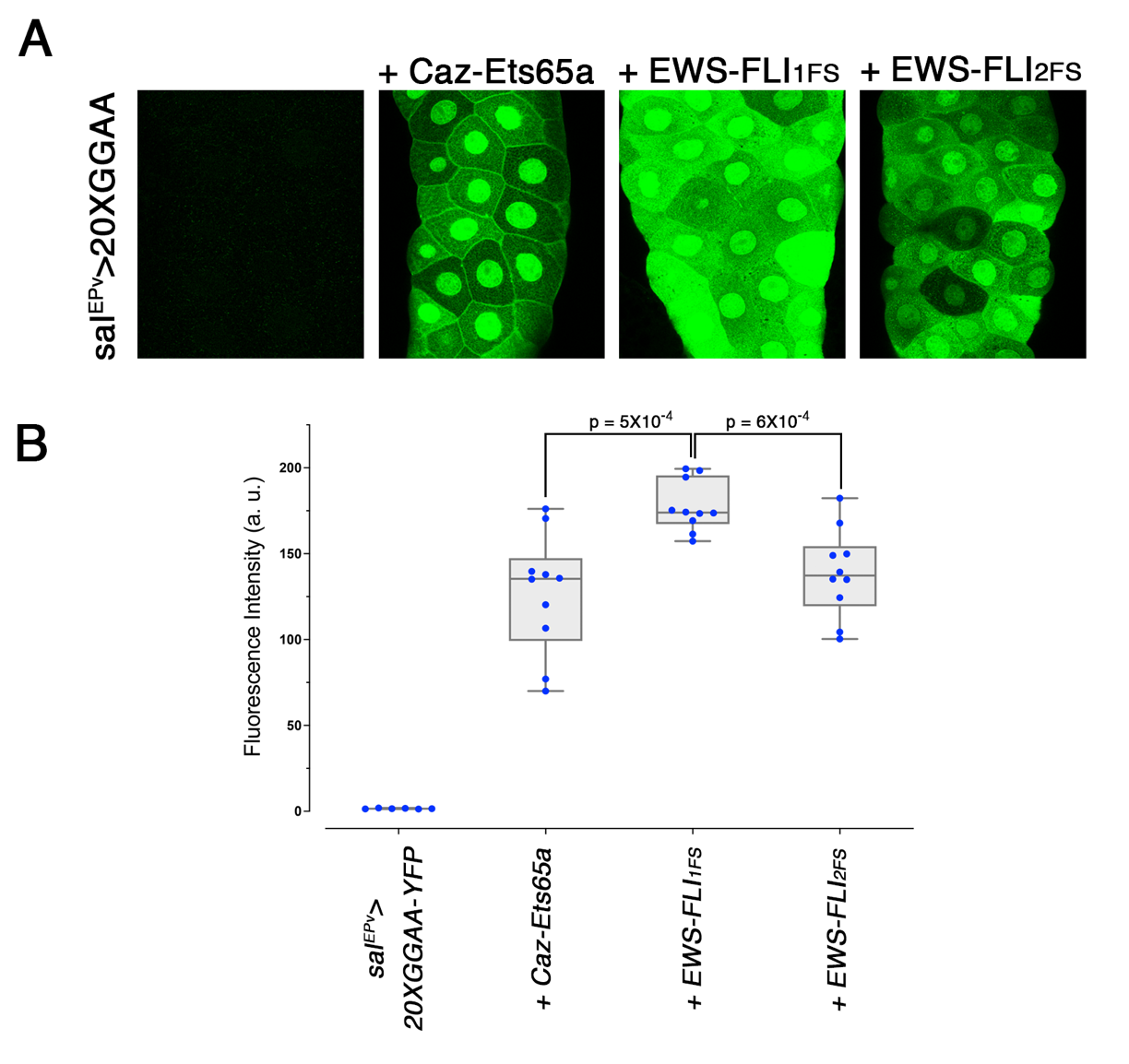


Fig. S8. Activation of 20X(GGAA)µSat-YFP by EWS-FLI1FS, EWS-FLI2FS and Caz-Ets65a

**(A)** Salivary glands from control (*sal^EPv^-Gal4>20X(GGAA)µSat-YFP*), Caz-Ets65a (*sal^EPv^-Gal4>UAS-Caz-Ets65a; 20X(GGAA)µSat-YFP*)*,* EWS-FLI1FS (*sal^EPv^-Gal4>UAS-EWS-FLI1FS; 20X(GGAA)µSat-YFP*) and EWS-FLI2FS (*sal^EPv^-Gal4>UAS-EWS-FLI2FS; 20X(GGAA)µSat-YFP*) third instar larvae showing YFP expression (green). **(B)** Quantification of YFP fluorescence intensity in control, Caz-Ets65a*,* EWS-FLI1FS and EWS-FLI2FS salivary glands. Levels of YFP fluorescence intensity are significantly higher in EWS-FLI1FS than in Caz-Ets65a, or than in EWS-FLI2FS salivary glands (unpaired *t* test; *p* < 0.001). a.u., arbitrary units.


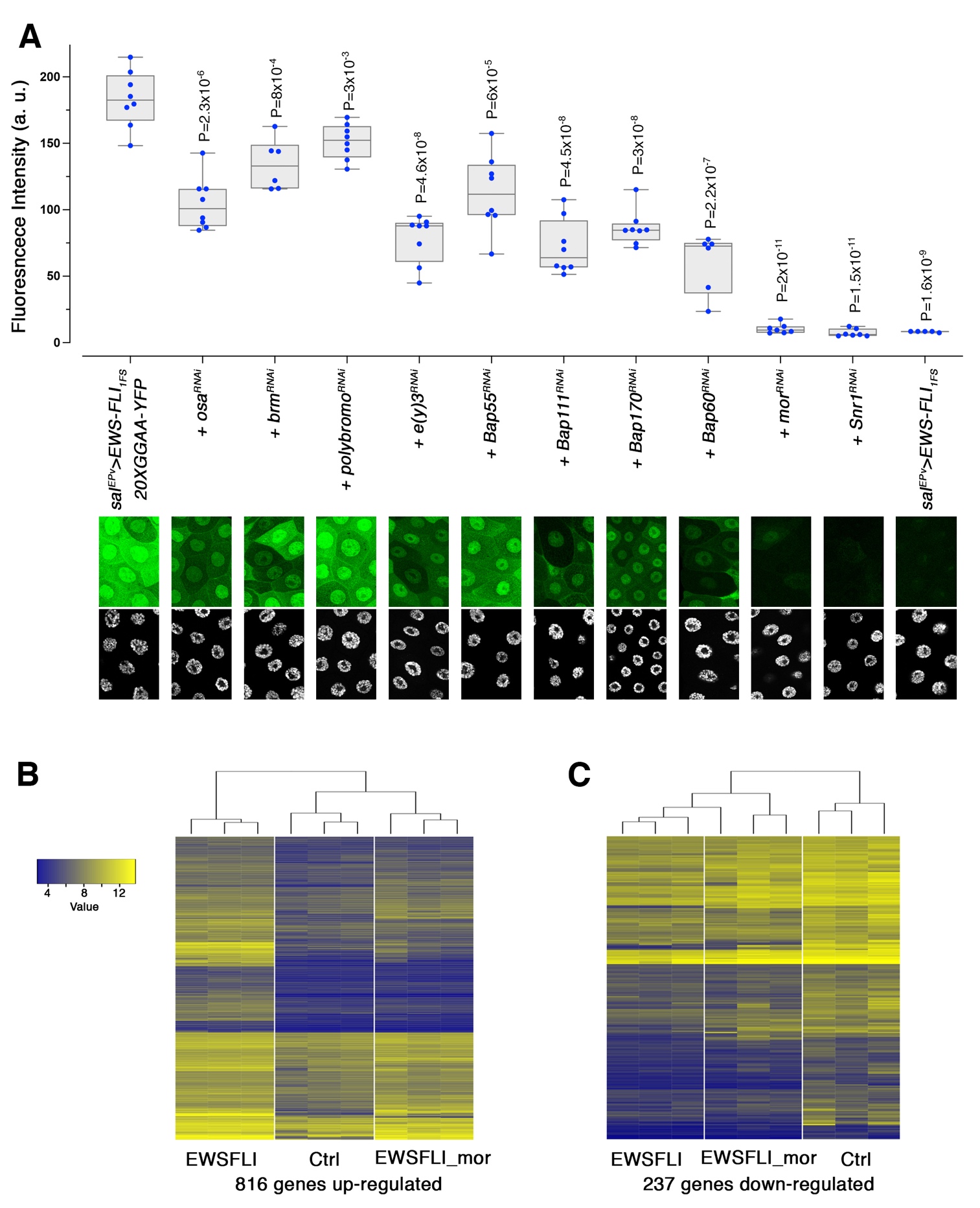


Fig. S9. BAP-dependent GGAAµSats trancription.

(**A**) Quantification of YFP fluorescence intensity in *sal^EPv^-Gal4>UAS-EWS-FLI1FS* salivary glands carrying *20X(GGAA)µSat-YFP* transgene. Depletion of either of the ten members of the Drosophila BAP/BAF complex significantly reduced the EWS-FLI-dependent YFP expression (unpaired *t* test; *p* < 0.01). Below are representative examples of YFP (green, upper panels) and DAPI staining (grey, lower panels) of salivary glands. a.u., arbitrary units. **(B-C)** Heatmaps of gene expression profiles of control (Ctrl; *sal^EPv^-Gal4/+*), EWS-FLI (*sal^EPv^-Gal4/UAS-EWS-FLI_1FS_*), and EWS-FLI_mor (*sal^EPv^-Gal4 UAS-EWS-FLI_1FS_/mor^RNAi^*) salivary glands. Probesets correspond to genes significantly up (B) or downregulated (C) in EWS-FLI_1FS_ compared to Ctrl. Expression levels are reported as unscaled values; blue and yellow indicate low and high expression, respectively. Dendrograms on the top of the heatmaps show hierarchical clustering between samples.

**Table S1**. **GAL4s drivers used in this study to ectopically express EWS-FLI**.

| ***GAL4*** | **Expression** | **Viability** |
| --- | --- | --- |
| *da-GAL4* | Ubiquitous expression | Embryonic Lethal |
| *Ubi-Gal4* | Ubiquitous expression | Embryonic Lethal |
| *tub-GAL4* | Ubiquitous expression | Embryonic Lethal |
| *act5C-Gal4* | Ubiquitous expression | Embryonic Lethal |
| *twi-GAL4* | Mesoderm | Embryonic Lethal |
| *Mef2-GAL4* | Mesoderm | Embryonic Lethal |
| *48Y-GAL4* | Endoderm | Embryonic Lethal |
| *D42-GAL4* | Neuromuscular junctions | Embryonic Lethal |
| *ogre-GAL4* | Larval CNS - Neuroepithelium | Early Pupal Lethal |
| *longGMR-GAL4* | Eye discs (cells posterior to the morphogenetic furrow (MF)) and optic lobes during late larval and early pupal stages | Viable  Rough eye and loss of pigmentation |
| *ey-GAL4* | Eye disc | Larval/Early Pupal Lethal  Headless pupae |
| *en-GAL4* | Posterior compartment of embryonic segments and larval imaginal discs. | Embryonic Lethal |
| *sal^EPv^-Gal4* | Wing disc (wide band centered on the anterior-posterior compartment of the wing pouch) | Viable  Abnormal adult wing patterning |
| *nub-Gal4* | Wing disc (pouch and hinge). CNS | Early Pupal Lethal  Tumorous wing disc |

Note: *GawB*-derived elements display constitutive expression of GAL4 in larval salivary glands (72, 73).

Table S2. EWS-FLI1FS transcriptomic signature.

List of genes significantly up (FC>2) and downregulated (FC<-2) in nub>EWS-FLI_1FS_ salivary glands.

Table S3. Primers used in this study.

| **Primers for cloning (5’-3’)** | | | |
| --- | --- | --- | --- |
| HSPGFPEI rev | | CACCATTTTGGAATTCCCAATTCCC | |
| HSP fw | | AACGGAGACTCTAGCGAGCGCCGGAG | |
| 3XGGAAHSP fw | | TAGCGGATCCAAGCTTGCATGCCTGCAGGTGAGGGAGGAAGGAAGGAAAAGAAACGGAGACTCTAGC | |
| 3XGGAAHSP rev | | GCTAGAGTCTCCGTTTCTTTTCCTTCCTTCCTCCCTCACCTGCAGGCATGCAAGCTTGGATCCGCTA | |
| 10X3XGGAA HSP fw | | TAGCGGATCCAAGCTTGCATGCCTGCAGGTGAGGGAGGAAGGAAGGAAGGAAGGAAGGAAGGAAGGAAGGAAGGAAAAGAAACGGAGACTCTAGC | |
| 10X3XGGAAHSP rev | | GCTAGAGTCTCCGTTTCTTTTCCTTCCTTCCTTCCTTCCTTCCTTCCTTCCTTCCTTCCTCCCTCACCTGCAGGCATGCAAGCTTGGATCCGCTA | |
| 1xEts65aHSP fw | | TAGCGGATCCAAGCTTCCGGAAGTAACGGAGACTCTAGCGAGCGCCGGAG | |
| T1-EcoRI | | ATAGGGAATTGGGAATTCCAAAATGGCC | |
| T1-XhoI | | TCTAGAGGTACCCTCGAGTCAGTAGTAG | |
| T1-FS fw | | ACAAGTACCCGTCCGAGCTACCATGCCCACCAGCAGAAG | |
| T1-FS rev | | CGGACGGGTACTTGTACATGCTGGAC | |
| T1-delSTOP | | TCTAGAGGTACCCTCGAGTGAGGACGGGTACTTGTACATGCTGGAC | |
| T2-EcoRI | | ATAGGGAATTGGGAATTCCAAAATGGCT | |
| T2-FS rev | | GCTCGGGTACTTGTACATGGAGGAC | |
| T2-delSTOP | | CTCTAGAGGTACCCTCGAGTCAGCTCGGGTACTTGTACATGGAGGAC | |
| T2T1-FS fw | | TACAAGTACCCGAGCGAGCTACCATGCCCACCAGCAGAAG | |
| FUS-EcoRI-UP | | GAATAGGGAATTGGGAATTCCAAAATGGCCAGCAA | |
| EWS-EcoRI-UP | | GAATAGGGAATTGGGAATCCAAAATGGCCTCCAC | |
| FEV-delta-XhoI | | CTAATCTCGAGTCAGGCGGGCAGCTTGTACAGGGC | |
| ERG-delta-XhoI | | TTGAATCTCGAGTCAGCTGGGGTACTTGTACAGGC | |
|  |  | | |
| **Primers for RT-qPCR (5’-3’)** | | | |
| **Gene** | **Forward** | | **Reverse** |
| *DAT* | CGGTGGCGCTTTTCTTGTG | | CCCAGGGCCAATTCCATATAGA |
| *Ilp2* | AAAGCTCAACGAGGTGCTGA | | CTGTCGGCACCGGGCAT |
| *CG9813* | GAGGAATCTGTTGGCCATCCT | | TGCGCTTGGAAGGATTGTAG |
| *CG31106* | GAACTCAATGGATAAGGCGGTC | | CAGGTAGCCCCAAAAGTATGATG |
| *prc* | GCAAGCGCAAATGGAGCTG | | CCCTCGAATAGCCTCTGCC |
| *Gsc* | CCTGCATCTGGGTCACTTGG | | TTGGGGTACGCCACCAAAAA |
| *CG10853* | TGACTGGAGCTGAGATGAGGA | | GCTGCTCCCAGGGATTATTGT |
| *CG12910* | GACTCAGTCACCTTAGTCCTACC | | GGTCGAGATCGGCCAAACAT |
| *Tsp5D* | GGCTACACTTGCATACGCC | | TCCTAAAAACGCACAGCTACAT |
| *Sgs3* | CTACCGCCCTAGCGAGC | | TAGTTGTAGTTGTGGGGCATCC |
| *Sgs1* | TCGCCCTTATCTTTTTAACTGTGAG | | TCACTGGGACCATCTTGCAT |
| *CG31698* | GCTGGATATGCCCGTCTTCT | | CCGCATTTGTAATTCATGCCCT |
| *CG7402* | ATGTTCCGAACCTATGCACAC | | CCACGGCTCATCGGTGATAAT |
| *cln3* | CAGACGTGCTGCCCTCATTA | | AGAGCTATACGGAAATTCACCCA |
| *net* | TTTACATGCAACTAAGCGCATCG | | GCATCACGGTCGTTGGAATTG |
| *CG7512* | CACCCAGATCACCTCCATCG | | TGGGCGCTCTTGTAGACCT |
| *Eig71Eg* | GGAGGAAATTAACCCGTTGTGA | | CGGCAACATTTTTGCAGGACC |
| *RpL32* | TGCTAAGCTGTCGCACAAATG | | CGTAACCGATGTTGGGCATC |
| **Primers for RT-qPCR (5’-3’)** | | | |
| **Gene** | **Forward** | | **Reverse** |
| *GADPH* | GGTCTCCTCTGACTTCAACA | | GTGAGGGTCTCTCTCTTCCT |
| *HPRT1* | CATTATGCTGAGGATTTGGAAAGG | | CTTGAGCACACAGAGGGCTACA |
| *NKX2-2* | CAGCGACAACCCGTACAC | | GACTTGGAGCTTGAGTCCTGA |
| *NR0B1* | AGGGGACCGTGCTCTTTAAC | | CTGAGTTCCCCACTGGAGTC |
| *PPP1R1A* | CACAGAAGTGGAGTCAAGGCTG | | TTGGCTCCCTTGGAATCCAGTG |
| *EWS-FLI1* | AGCAGCCTCCCACTAGTTAC | | CCAAGCTCCTCTTCTGACTG |
| *SOX2* | TTGCTGCCTCTTTAAGACTAGGA | | TAAGCCTGGGGCTCAAACT |
| *FCGRT* | TGGCGATGAGCACCACTAC | | GATTCCCACCACGAGCAC |

SI References

65. M. Calleja, E. Moreno, S. Pelaz, G. Morata, Visualization of gene expression in living adult Drosophila. *Science* **274**, 252-255 (1996).

66. C. Cruz, A. Glavic, M. Casado, J. F. de Celis, A gain-of-function screen identifying genes required for growth and pattern formation of the Drosophila melanogaster wing. *Genetics* **183**, 1005-1026 (2009).

67. F. Rossi, C. Gonzalez, Studying tumor growth in Drosophila using the tissue allograft method. *Nat Protoc* **10**, 1525-1534 (2015).

68. J. Bischof, R. K. Maeda, M. Hediger, F. Karch, K. Basler, An optimized transgenesis system for Drosophila using germ-line-specific φC31 integrases. *Proceedings of the National Academy of Sciences* **104**, 3312-3317 (2007).

69. M. Giovannini *et al.*, EWS-erg and EWS-Fli1 fusion transcripts in Ewing's sarcoma and primitive neuroectodermal tumors with variant translocations. *J Clin Invest* **94**, 489-496 (1994).

70. M. Peter *et al.*, A new member of the ETS family fused to EWS in Ewing tumors. *Oncogene* **14**, 1159-1164 (1997).

71. H. Ichikawa, K. Shimizu, Y. Hayashi, M. Ohki, An RNA-binding protein gene, TLS/FUS, is fused to ERG in human myeloid leukemia with t(16;21) chromosomal translocation. *Cancer Res* **54**, 2865-2868 (1994).

72. O. Gerlitz, D. Nellen, M. Ottiger, K. Basler, A screen for genes expressed in Drosophila imaginal discs. *Int J Dev Biol* **46**, 173-176 (2002).

73. B. F. Costantino *et al.*, A novel ecdysone receptor mediates steroid-regulated developmental events during the mid-third instar of Drosophila. *PLoS Genet* **4**, e1000102 (2008).
